# Applying model approaches in non-model systems: A review and case study on coral cell culture

**DOI:** 10.1371/journal.pone.0248953

**Published:** 2021-04-08

**Authors:** Liza M. Roger, Hannah G. Reich, Evan Lawrence, Shuaifeng Li, Whitney Vizgaudis, Nathan Brenner, Lokender Kumar, Judith Klein-Seetharaman, Jinkyu Yang, Hollie M. Putnam, Nastassja A. Lewinski

**Affiliations:** 1 Life Science and Engineering, Virginia Commonwealth University, Richmond, Virginia, United States of America; 2 Department of Biological Sciences, University of Rhode Island, Kingston, Rhode Island, United States of America; 3 Aeronautics and Astronautics, University of Washington, Seattle, Washington, United States of America; 4 Department of Chemistry, Colorado School of Mines, Golden, Colorado, United States of America; Universitat Konstanz, GERMANY

## Abstract

Model systems approaches search for commonality in patterns underlying biological diversity and complexity led by common evolutionary paths. The success of the approach does not rest on the species chosen but on the scalability of the model and methods used to develop the model and engage research. Fine-tuning approaches to improve coral cell cultures will provide a robust platform for studying symbiosis breakdown, the calcification mechanism and its disruption, protein interactions, micronutrient transport/exchange, and the toxicity of nanoparticles, among other key biological aspects, with the added advantage of minimizing the ethical conundrum of repeated testing on ecologically threatened organisms. The work presented here aimed to lay the foundation towards development of effective methods to sort and culture reef-building coral cells with the ultimate goal of obtaining immortal cell lines for the study of bleaching, disease and toxicity at the cellular and polyp levels. To achieve this objective, the team conducted a thorough review and tested the available methods (i.e. cell dissociation, isolation, sorting, attachment and proliferation). The most effective and reproducible techniques were combined to consolidate culture methods and generate uncontaminated coral cell cultures for ~7 days (10 days maximum). The tests were conducted on scleractinian corals *Pocillopora acuta* of the same genotype to harmonize results and reduce variation linked to genetic diversity. The development of cell separation and identification methods in conjunction with further investigations into coral cell-type specific metabolic requirements will allow us to tailor growth media for optimized monocultures as a tool for studying essential reef-building coral traits such as symbiosis, wound healing and calcification at multiple scales.

## Introduction

Model organisms have delivered breakthroughs and new insights into key biological processes [1]. In mammalian and terrestrial systems humans, mice, *Caenorhabditis elegans* (roundworm), *Drosophila melanogaster* (common fruit fly), *and Aradopsis* (rockcress) have provided us a wealth of insight into gene structure and function [2,3], disease and immunity [4–6], and genome to phenome mapping [7,8]. The abundance of resources available through model system approaches has the capacity to catalyze research advances at a time of critical need as organisms worldwide are impacted by global stressors of the Anthropocene [9].

The opportunities to use model system approaches become less frequent as one moves from mammalian vertebrate models to aquatic and marine invertebrates that are ecologically important. There is a growing number of models, however, for cnidarians given their basal location on the tree of life and ecological and economic importance in coral reef ecosystems. In the aquatic world, for example, *Hydra* and *Nematostella* (starlet sea anemone) have been used to generate cnidarian genomes, and Aiptasia (tropical sea anemone; *Exaiptasia spp*.) to study gene knockdown by RNAi, symbiosis and immunofluorescence [10] and references therein. *Exaiptasia diaphana (Exaiptasia pallida)* has generated the most traction in cnidarian model systems [11–16] with the capacity to harbor endosymbiotic dinoflagellates from four Symbiodiniaceae genera (*Symbiodinium*, *Breviolum*, *Cladocopium*, *Durusdinium*; [17] allowing comparative approaches to distinguish organismal functions according to symbiont identified. Several lines of evidence incorporating physiological [13], transcriptomic [18], proteomic [19], epigenetic [20], and metabolomic approaches [21,22] elucidate the tradeoffs associated with harboring different endosymbionts.

Coral reef ecosystems are specifically of interest in model development, as reef building corals are responding as a “canary in the coal mine” to climate change. While coral reefs worldwide support 25% of all marine life, rising concerns for their future persistence are intensifying [23,24]. Specifically, heat waves associated with rising ocean temperatures are disrupting the intricate and essential nutritional symbiosis between cnidarian hosts and their single celled dinoflagellate endosymbionts [25], in the family Symbiodiniaceae [26]. This symbiotic breakdown leads to a loss or expulsion of the photosynthetic algal cells [27], which results in the white skeleton appearing visible through the translucent coral tissues, in a detrimental phenomenon known as coral bleaching reviewed in van Oppen & Lough [28]. Mass coral bleaching events have increased in magnitude and frequency, with substantial loss of reef building corals in areas such as the Great Barrier Reef in Australia and other reefs worldwide [29,30]. This crisis highlights the need for model systems approaches to study the impacts of changing ocean environments on coral biology. Furthermore, it is clear that the handful of marine model systems already studied (e.g., urchin *Strongylocentrotus purpuratus* [31]; ascidian http://tunicate-portal.org/, [32]; oyster *Crassostera gigas*, http://gigaton.sigenae.org, [33]; and Squid *Euprymna scolopes* [34] are ill-equipped for addressing this need, as other models are more evolutionarily derived, or do not combine a primary nutritional symbiosis and calcification. A shift towards model systems approaches will be particularly impactful for threatened reef building corals and marine research as a whole [10,35].

Existing resources used to study genomics and their developmental biology will serve as important tools to turn reef-building corals into model cnidarian taxa [e.g. 36,37]. Traits such as the potential to exist in multiple symbiotic states (symbiotically and aposymbiotically) living sympatrically (e.g. *Astrangia poculata*, aposymbiotic *Astrangia* do not harbor symbionts whereas their symbiotic counterparts harbor *Breviolum psygmophilum*, [38,39] will allow the study of facultative symbiosis ranging from cellular, to organismal, to ecological approaches [38,40,41]. Although scleractinian corals are considered non-model systems, they manifest many qualities of model systems. Indeed, scleractinian corals, as clonal and colonial organisms, represent a readily available source of clonal population [10], a certain number of species manifest high phenotypic plasticity [42], and have been intensively studied both in field and laboratory settings [43–48]. Scleractinian corals are also known for harboring not only symbiotic dinoflagellate algae but also a diverse microbiome [49,50], including bacteria and viruses common to other life forms [51]. Further comparative physiology, transcriptomics, and the growing number of assembled genomes [52–55] contribute to the development of stony corals as model organisms. Study of these taxa with model systems approaches will not only improve our understanding of cnidarian biology, but also aid in the management and restoration practices for these valuable [56,57] ecosystem engineers.

To date, some scleractinian corals that have been suggested as potential model species include: *Acropora millepora and Stylophora pistillata* for the Indo-Pacific based on regional abundance, the ease with which they can be sampled (branching corals) and reared in aquaria, and existing genomic resources [55,58,59], *Acropora palmata* in the Caribbean because of its endangered status [10,60–62], *Montipora capitata* in Hawaii due to its endemic status, unique genome architecture, and its plasticity in performance when hosting primarily *Cladocopium spp*. or *Durisdinium glynnii* symbionts [63–65], and *Pocillopora damicornis* for its abundance and widespread geographical distribution [53,66,67]. Considering the aforementioned criteria in combination with calcification, symbiosis, and thermal tolerance, other species can be identified amongst the vast diversity of reef corals. The real lynchpin of successful model approaches are not the species chosen, but primarily the capacity that methods such as polyp scale models, immortal coral cell lines, closed life cycle, genomic resources, and an open and engaged community of researchers provide. Together this foundation will support the application of ‘omic techniques [68–70], and functional genetic analysis such as CRISPR [71] to help us elucidate the still unknown aspects of these invaluable metaorganisms [68].

As studies embrace the importance of simulating environmentally-relevant conditions (e.g. pH and temperature fluctuations; [72,73], controlled laboratory testing is also necessary to deconvolute variables and directly mechanistically link the impact of a specific environmental change, such as presence of contaminants, to changes in coral health. Useful as they are, traditional model organisms alone are limited in their capacity to address questions pertaining to the response to climate change at the cellular level, to biomineralization, or to the microbial symbioses [10,51,74]. For this, methods for immortal cell lines, cell sorting [75], and single cell analyses [e.g. 76] need to be developed and/or advanced.

Free-living populations of isolated symbiotic cells (dinoflagellates from the family Symbiodiniaceae) have been successfully maintained and studied in culture collections since the late 1950s-early 1960s [77], but not all can be successfully cultivated [78]. From the host perspective, naturally, the two cell types that have received the most attention are the gastrodermal cells that can contain symbiotic dinoflagellate e.g. [27,77–82], and the cells responsible for biomineralization, the calicoblastic cells [79,80,83–85]. Recent work in the starlet anemone *Nematosella vectensis* and the soft coral *Xenia sp*. are the first to assess gene expression in cnidarians using a single cell RNASeq approach, and have provided detailed gene expression signatures for eight cnidarian cell classes and subtypes [86], and identified a putative endosymbiosis gene set [76], which will be essential for further functional testing. Our knowledge of the biomineralization cells and process are relatively more advanced than endosymbiotic cells, with a putative biomineralization toolkit [87,88] and multiple sequenced proteomes for the skeletal organic matrix [87,89]. To date however, neither coral gastrodermal cells nor calicoblastic epithelial cells have been successfully isolated and grown as immortal cell lines, hampering functional work that would improve our understanding of these essential processes.

Inverted microscopy, with live imaging directed at the growing edge of coral fragments or polyps on glass slides has, to date, been the most effective way of investigating coral calcification and carbonate chemistry of the extracellular calcifying fluid at high magnification [90–92] along with tissue balls and “proto-polyps” [84,93,94]. The Fluorescence Activated Cell Separation (FACS) method adapted by Traylor-Knowles and colleagues [75,95] to coral cells allows sorting and identification of different cell populations, and has potential for immunological studies. Nevertheless, there is a huge gap in our understanding of cellular and molecular processes in corals largely because coral cells are difficult to maintain and grow *in vitro* under controlled environmental conditions over extended periods of time [80,83,93,96,97]. Hence, there is a global need to establish rigorous cell-based culture methods for a variety of cell types to understand the molecular mechanism associated with coral cell biology and functions. However, establishing cell-based systems is challenging due to the limited information available on the complex interactions between cells, tissues, the microbiome and the holobiont equilibrium.

The work presented here reviews and assesses methods reported to date on dissociating and culturing coral cells as an initial step towards the overarching goal of obtaining immortal cell lines for the study of bleaching, disease and toxicity at the cellular and polyp levels. To better compared the available cell culture methods, we conducted tests of a set of methods across laboratories on a single coral genotype. The coral species choice (*Pocillopora acuta*) was mainly guided by the availability of many samples from the same coral genotype and the amount of existing data however, we did not limit our review and assessment of approaches to only those reported for that species. By examining methods applied to various cnidarian species, we aimed to identify cell culture techniques that can be applied to a variety of scleractinian corals, to place them amongst model systems approaches, and generate significant research advances in the field.

## Materials and methods

### Aquaculture

#### Pocillopora acuta

The species used here is *Pocillopora acuta* (Lamarck, 1618). Specimens from the same genotype were purchased from Ocean State Aquatics (Coventry, RI), sequence details in S1 File. The *Pocillopora* genus has suffered from frequent species misidentification due to high levels of phenotypic plasticity [42,98]. More specifically, *Pocillopora damicornis* and *P*. *acuta* show signs of potential hybridization or incomplete lineage sorting which has led to confusion and mistakes in reporting [98]. The diversification of extant *Pocillopora* species originated from a common ancestor within the last ~3 million years [98]. *P*. *acuta* is a hermaphroditic species of scleractinian coral that manifests mixed reproduction methods [98,99], and represents, with *Pocillopora damicornis*, one of the most extensively studied species of reef building corals as a consequence of its wide geographic distribution [100]. *P*. *acuta* can be found in shallow tropical to subtropical waters of the Pacific Ocean, throughout Southeast Asia, the Indian Ocean and the Red Sea. This species equally grows in sheltered or exposed reef habitats, upper reef slopes, and as deep as 40m [100]. Growth rate and branching morphology depend greatly on environmental conditions [42] but its common designations, cauliflower coral and lace coral, give indications about its general shape.

#### Aquaria

Coral fragments were maintained in a 37.8L glass aquarium containing artificial seawater (Specific Gravity 1.025 ±0.002, 30-36ppt salinity, Instant Ocean Reef Crystals) heated to 24–25°C and illuminated using a AI^®^ Prime^™^ 16HD Smart Reef LED lighting system (8 LED colors, set to generate ~30% PAR at coral level) on a 10 h light: 14 h dark cycle. Water flow of 378.5 L/h was maintained using a filter system. Water chemistry was tested weekly (API 5 in 1 test strips: pH, NO_2_^-^, NO_3_^-^, KH, GH) to verify maintenance of pH (8 ±0.5), nitrogen sources (~0), and dissolved inorganic carbon (>80ppm) levels. While certain laboratory setups differed slightly, the key parameters (light, seawater temperature, salinity, pH, flow) were kept consistent between each laboratory part of this study.

### Cell dissociation

Different methods for separating the coral tissue from the skeleton were tested, from scraping with a surgical scalpel or hook, to using a simple brush (paint brush or toothbrush) or calcium-, magnesium-free seawater incubation. Yield and survival being critical, methods inducing minimum cell damage should be preferred. The yield of brushing methods can vary depending on the applied pressure and rigidity of the bristle material. The use of calcium-, magnesium-free seawater [83] has been reported to result in spontaneous detachment of coral tissue from the underlying skeleton. This method relies on the fact that calcium and magnesium are known to promote cell adhesion and, by withholding these elements, tissue and cell detach from the skeleton. This method has been reported to produce both isolated cells and cell aggregates [83]. The full method description can be found in S2 File.

Enzymatic digestion is a commonly used process of non-mechanical tissue and cell dissociation, i.e. separating cells from each other. The enzymes involved target cell-cell and cell-extracellular matrix bonds [97,101] and each method requires adjusting treatment parameters to cell type. This may involve increasing the tissue surface to provide a large contact surface area for enzyme activity, and enzymatic digestion of extracellular matrix components and cleaving cell-cell contacts. Cell damage should be kept to a minimum to avoid cellular DNA contamination. The enzymes tested here are trypsin (Fisher scientific #25300054) and a combination of trypsin, liberase with collagenase (Millipore Sigma #5401119001). The full method description can be found in Supplementary material: S1 Table. Briefly, 2 mL of trypsin (concentrations tested: 0.125% and 0.025% concentration) were added to the coral cell pellets and incubated for 5 min minimum at 25°C; 2 mL of trypsin (concentrations tested: 0.125% and 0.025% concentration) mixed with 2 mL of liberase with collagenase solution were added to coral cell pellets and incubated for 3 days at 25°C. In both cases, culture media was added to neutralize enzyme digestion and subsequently centrifuged.

### Cell sorting

The methods explored to efficiently separate different cell populations for monocultures are density gradient centrifugation [102], fluorescence assisted cell sorting [FACS, 73,74,93]. The density gradient centrifugation relies on cell type specific densities while the FACS method uses, in addition to endogenous fluorescence, fluorescent proves to separate the cell populations using a FACS machine. The tests performed using FACS relied on the methods (protocol, staining and gating) developed by Rosental et al. [75]. The full description of the FACS method can be found in S2 File.; briefly, coral cell suspension aliquots mixed with DAPI working solution (DAPI + phosphate-buffered saline) are run through the FACS machine (BD FACSAria ^™^ II High-Speed Cell sorter with BD FACSDiva Software). The full description of the density gradient centrifugation (Percoll) method can be found in S2 File; briefly, 1 mL of Percoll solution (concentrations at 100, 90, 80, 70, 60, 50, 40, 30, 20, 10, and 5% concentration) were added to 2 mL of coral suspension and centrifuged for 10 min at 1460 rpm.

### Factors of growth

A combination of culture media reagents, antibiotics (Penicillin-Streptomycin, Antibiotic-Antimycotic, Gentamicin) and artificial seawater was tested using Dulbecco’s Modified Eagle Medium (DMEM) and Roswell Park Memorial Institute medium (RPMI, mammalian cell culture medium). DMEM and RPMI are common basal culture media for mammalian cell cultures and have been used for coral cell cultures in previous works [e.g. 79,81,94,100]. The full protocol can be found in Supplementary material S2 File; briefly, 74 mL of artificial seawater (details S2 File) were mixed with 15 mL of DMEM (Fisher Scientific #30243010), 10 mL of Fetal Bovine Serum (FBS, Thermofisher Scientific #26140087), 1 mL of Antibiotics-Antimycotics solution (Anti-Anti, composed of Penicillin, Streptomycin and Amphotericin B, Thermofisher Scientific #1524006) and 0.5 mL of Gentamicin (Thermofisher Scientific #15710064).

### Cell attachment

To promote cell adhesion, a number of surfaces were tested: plain glass, tissue culture treated (TCT) plastic, collagen coated glass and collagen coated TCT plastic.

Consideration of cell seeding density is important as the number of cells influences the response to ligands and xenobiotic materials in static batch experiments. Because different cells have different growth rates, seeding density also affects time to confluency, or complete cell coverage of the substrate. Coral cell diameters range between 10–25 microns [27,103] which is comparable to the cell diameters of human cells. Drawing parallels between human and coral cell culture, seeding densities of 15,000–30,000 cells/cm^2^ are optimal for imaging and seeding densities of 300,000–500,000 cells/cm^2^ are optimal for confluency at the onset of the experiment.

### Cytotoxicity

Trypan blue was used to test cell viability (live cells vs dead cells); 10 μL of 0.4% trypan blue stain (Millepore Sigma T1854) are added to 100 μL of coral cell suspension (1–2 x10^6^ cells/mL), blue cells represent the dead cells in a viable population. 10 μL of stained cell suspension was added to a hemocytometer for counting under a Nikon Eclipse TS100 Inverted Routine Microscope. The full counting protocol can be found in S2 File.

## Results

### Coral cell lines

**Fig 1:**
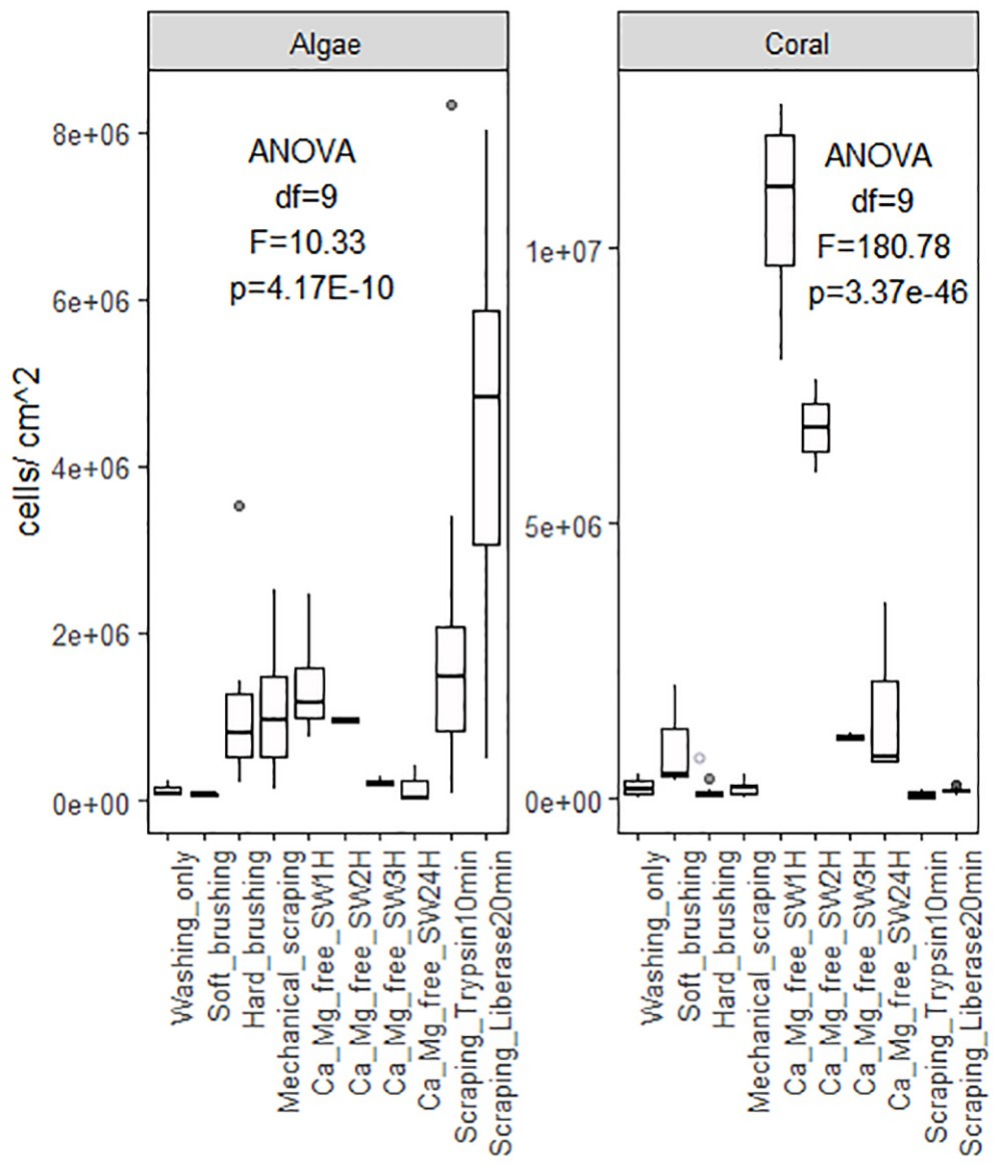
Cell yields according to dissociation method. Average algae cell (left) and coral cell (right) yields as a factor of dissociation method (washing, soft/hard brushing, mechanical scraping, or calcium-magnesium-free seawater incubation for 1 to 24 hours) and enzyme digestion (Trypsin or Liberase), S1 Table. [ANOVA single factor: Algae df = 9, F = 10.33, p = 4.17E-10; Coral df = 9, F = 180.78, p = 3.37E-46].

**Fig 2:**
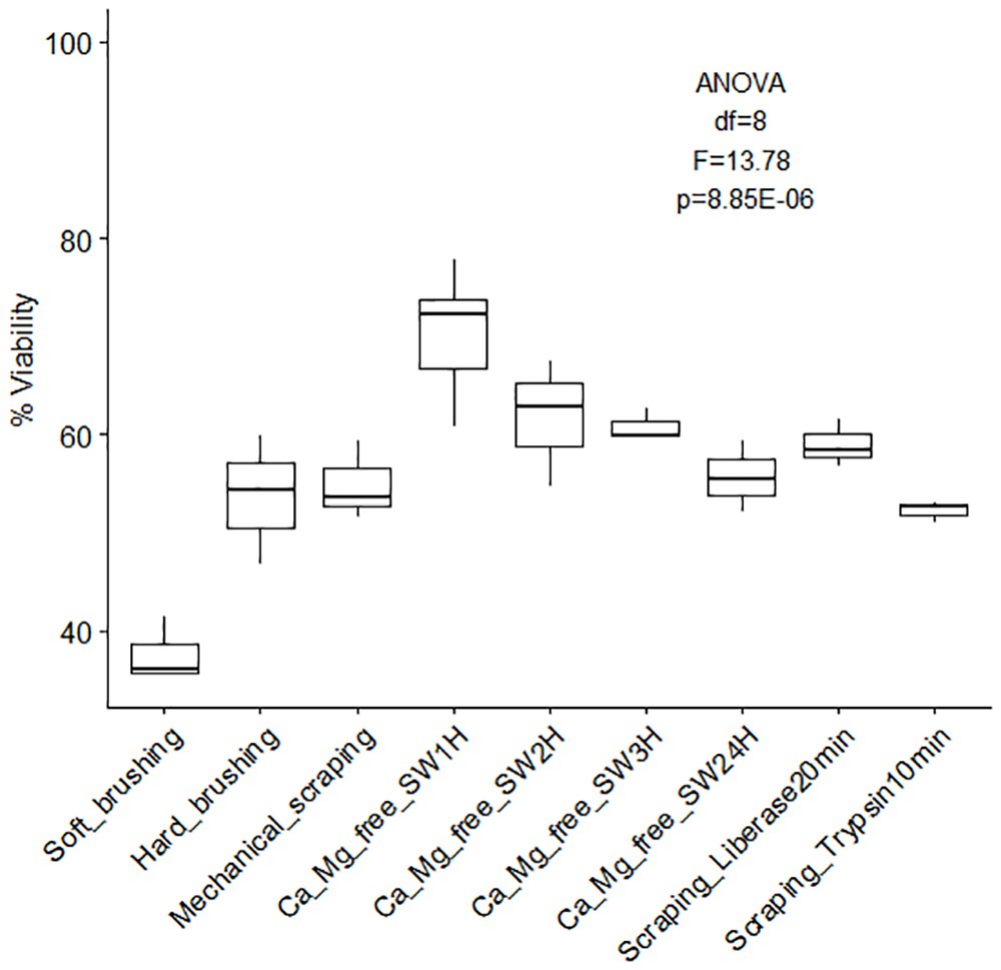
Overall cell viability (%) according to dissociation method. Percent viability (± SE) of cells dissociated from coral skeleton (immediately after dissociation) using different methods (soft/hard brushing, mechanical scraping, calcium-magnesium free seawater incubation for 1 to 24 hours, and enzyme digestion). S3 Table [ANOVA single factor: df = 8, F = 13.78, p = 8.85E-07].

**Fig 3:**
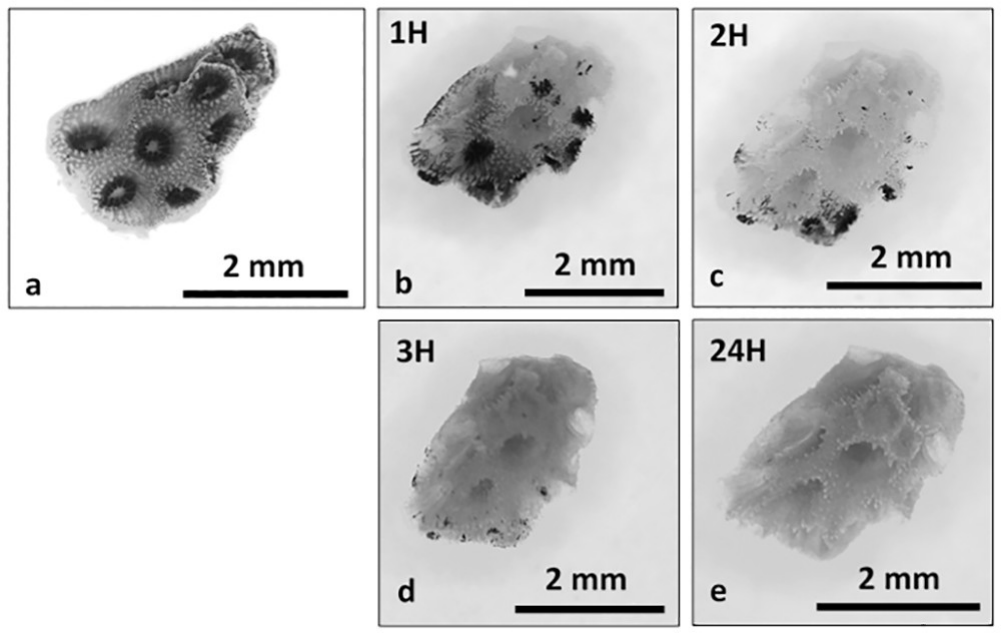
Ca^2+^-Mg^2+^ free seawater incubation. Time series photographs of Ca^2+^-Mg^2+^ free seawater coral nubbin incubation, T0 (a), 1H (b), 2H (c), 3H (d) and 24H (e). Photographs taken under a dissecting microscope.

Coral cell culture involves a series of steps for which different approaches can be used with varying results: cell dissociation (with or without isolation through enzyme digestion), cell sorting, cell attachment, and finally cell proliferation. Overall, the different methods tested to dissociated coral host cells and algae cells from coral nubbins yielded significantly different counts (algae cell yield ANOVA df = 9, F = 10.33, p = 4.17E-10; coral cell yield ANOVA df = 9, F = 180.73, p = 3.37E-47, Fig 1) and viabilities (ANOVA df = 8, F = 13.78, p = 8.85E-07, Fig 2). Amongst the four methods tested (washing, mechanical scraping, brushing and Ca^2+^-Mg^2+^ free seawater incubation), incubation in Ca^2+^-Mg^2+^ free seawater (Fig 3a–3e) for 1 hour yielded the maximum number of cells overall (1.39E+06 ±1.03E+06 algae cells/cm^2^, 1.07E+07 ±1.03E+06 coral cells/cm^2^, Fig 1 and S1 and S2 Tables) without enzyme digestion, and the highest average viability (70.4%, Fig 2, S3 Table). Both liberase and trypsin digestion in combination with scraping resulted in higher average algae yields (4.51E+06 ±4.58E+05 and 2.20E+06 ±8.29E+05 algae cells/cm^2^ respectively, Fig 1 and S1 and S2 Tables) compared to scraping alone (1.08E+06 ±1.39E+05 algae cells/cm^2^), but with high variability (±3.34E+06, n = 21, and ±3.96E+06, n = 9, respective 95% confidence interval of the mean, compared to ±1.78E+05, n = 9 hard brushing alone, Fig 1 and S1 Table). Focusing on coral cells only (i.e. host cells), Ca^2+^-Mg^2+^ free seawater incubation was most effective, especially 1 hour and 24 hours incubations (1H: 1.07E+07 ±1.03E+06 cells/cm^2^; 24H: 1.65E+06 ± 9.53E+05 cells/cm^2^). Enzyme digestion did not increase coral cell yield (Fig 1 and S1 and S2 Tables).

**Fig 4:**
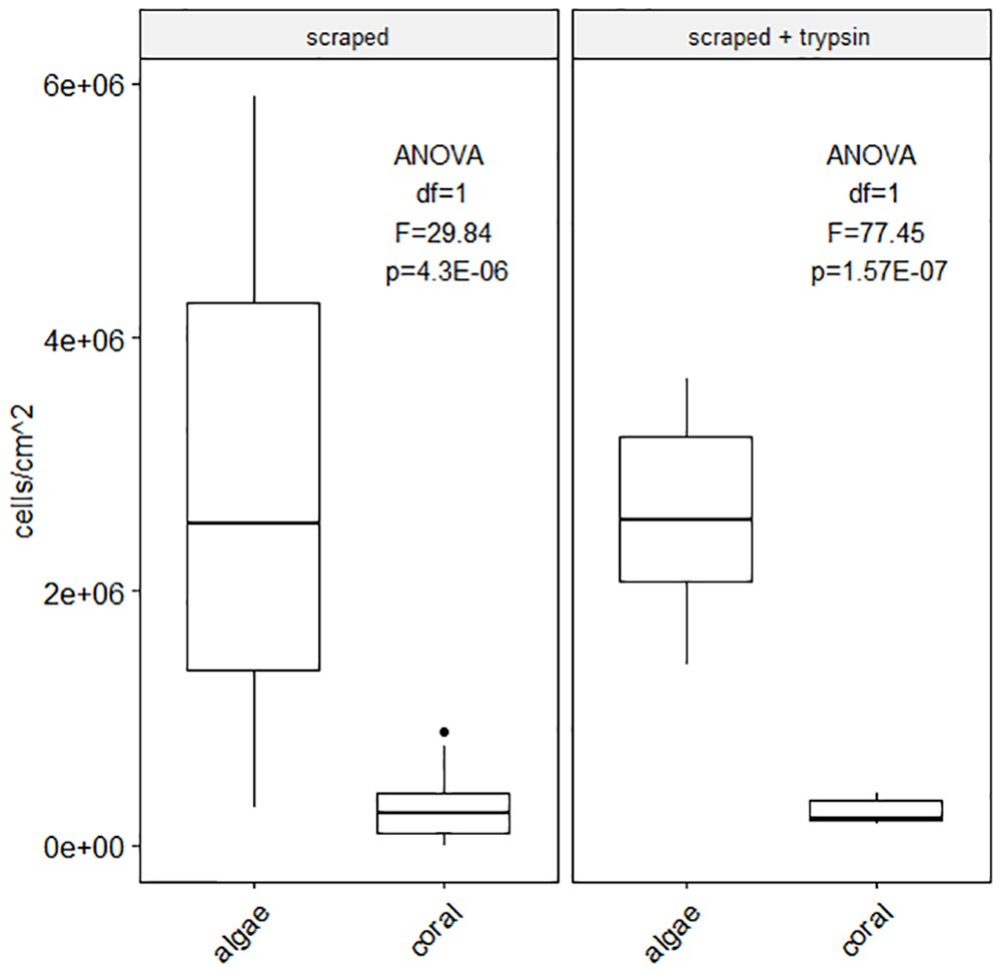
Cell dissociation method combination efficacy. Average algae cell and coral host cell yields as a factor of the origin of cell counted: Initial cell dissociation performed by scraping, remaining cells (on the skeleton) were dissociated using trypsin incubation for 1 hour. Significant difference between algae cell yield and coral cell yield by scraping and by scraping associated to trypsin digestion (scraped yield ANOVA df = 1, F = 29.84, p = 4.3E-06; scraped + trypsin yield ANOVA df = 1, F = 77.45, p = 1.57E-07) but no statistically different yields of algae cells and coral cells between methods (algae cell yield ANOVA df = 1, F = 0.057, p = 0.81; coral cell yield ANOVA df = 1, F = 0.48, p = 0.49) S3 Table.

To assess the amount of cells remaining after mechanical scraping, enzyme digestion was done on nubbins post-scraping (Fig 4). Cell yields revealed mechanical scraping dissociates only ~50% of cells with the remainder being dissociated using trypsin digestion post-scraping (scraped average: 2.75E+06 algae cells/cm^2^ and 2.93E+05 coral cells/cm^2^; scraped + trypsin: 2.59E+06 algae cells/cm^2^ and 2.57E+05 coral cells/cm^2^, Fig 4). Despite this, yields were not significantly different between simple scraping and scraping combined to trypsin digestion (algae cell yield ANOVA df = 1, F = 0.057, p = 0.81; coral cell yield ANOVA df = 1, F = 0.48, p = 0.49).

Two methods were tested to separate cell populations (qualitative approach): Percoll density gradient centrifugation (S2 File) and Fluorescence Activated Cell Separation (FACS, S2 File). The gradients created using Percoll were weak and layers were observed to leak into each other considerably. This method did not successfully separate different cell populations (S1 Table). FACS was successful at separating cell populations, such as aposymbiotic, symbiotic coral cells and additional sub-populations when using dyes, but the subsequent identification of these sub-population cell types needs to be improved before any quantitative data can be analyzed.

**Fig 5:**
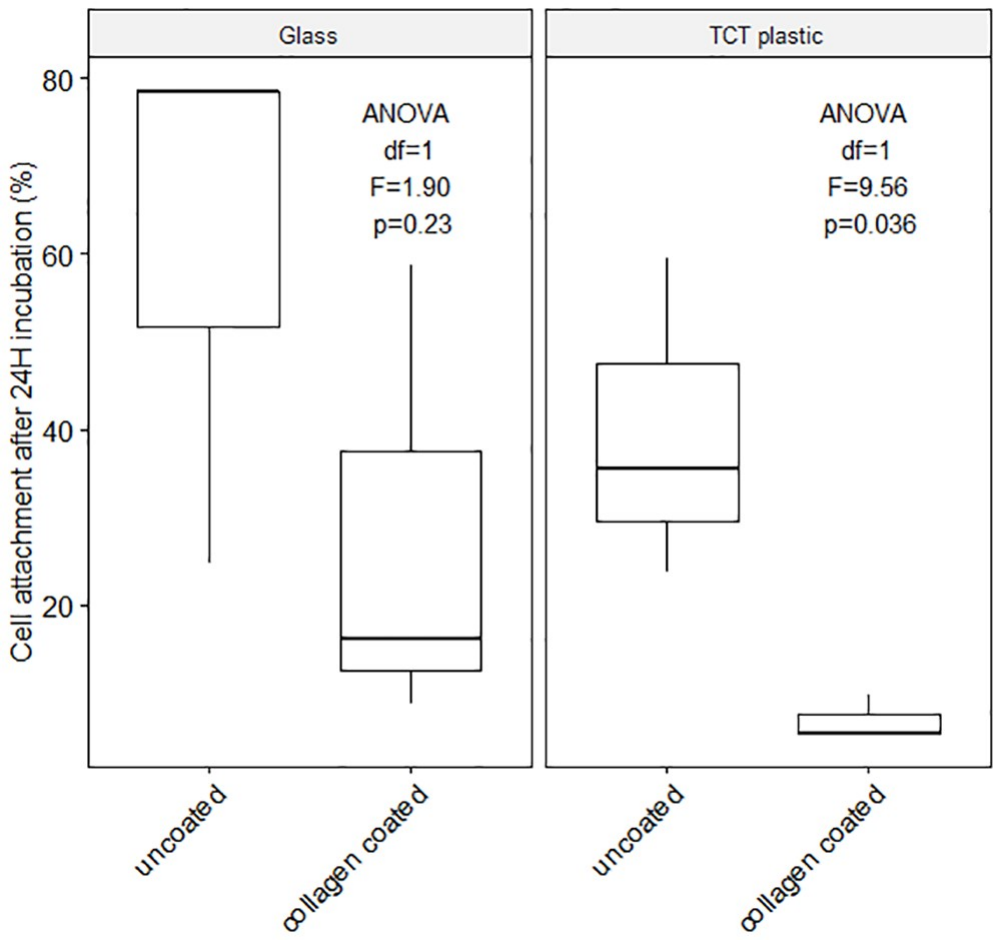
Cell attachment. Average cell attachment (%, miscellaneous *Pocillopora acuta* cells) on glass and tissue culture-treated (TCT) plastic, with and without collagen coating after 24H incubation at 25°C and 12 h light / 12 h dark cycle, S1 Table. (Initial cell dissociation: Ca^2+-^Mg^2+^ free seawater incubation for 1 hour; Culture medium combination use: 15% DMEM, 5% FBS, 1% Penicillin-Streptomycin, 79% sterile filtered artificial seawater, see S5 Table). Significant difference between coated and uncoated substrate (df = 1, F = 6.34, p = 0.036) but no significant difference between TCT plastic and glass (df = 1, F = 2.64, p = 0.143) S5 Table.

To exceed or at least match the longest coral culture duration (1 month, 94) adherent cells must attach to the culture substrate. To promote adhesion, two substrates with different treatments were tested with unsorted cell suspensions (S2 File): untreated glass, tissue culture treated plastic (TCT), collagen coated (Collagen coating solution, Sigma Aldrich Cat. No. 125–50) glass and collagen coated TCT plastic. The highest cell attachment was observed with untreated glass (60.64%) and tissue culture treated plastic (39.57%), Fig 5 and S5 Table, (glass ANOVA df = 1, F = 1.90, p = 0.23; TCT ANOVA df = 1, F = 9.56, p = 0.036). Lowest attachment was measured using collagen coated TCT plastic (6.83% attachment).

**Fig 6:**
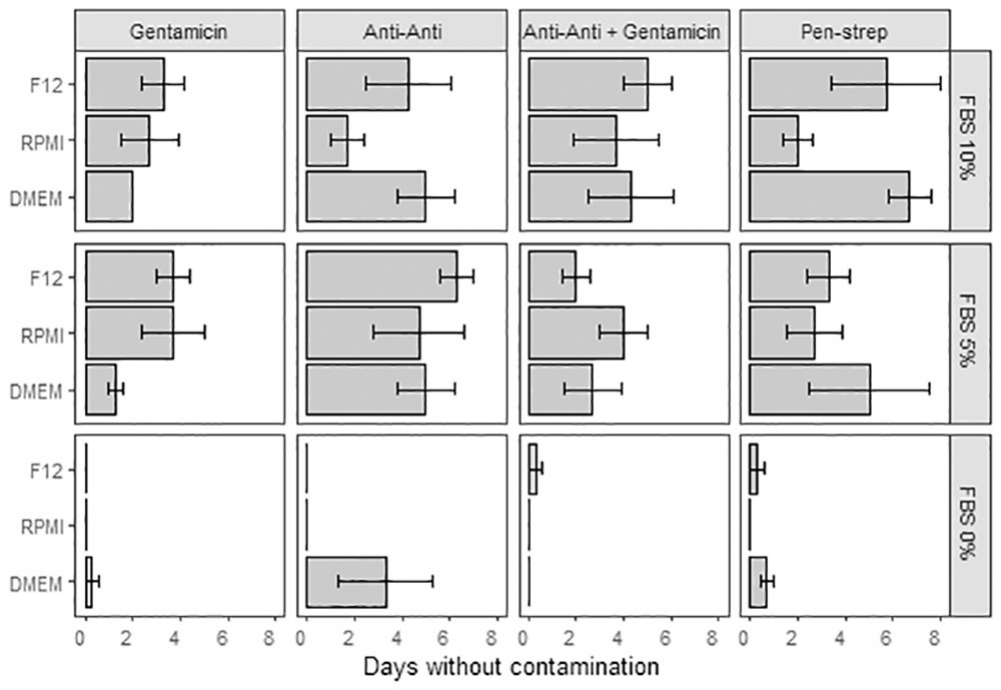
Cell culture contamination variations. Average contamination free cell culture duration (days ± SE) as a factor of media type (F12, RPMI, DMEM), serum (0%, 5%, 10%) and antibiotic (Gentamicin, Anti-Anti: Antibiotic-Antimycotic, Anti-Anti + Gentamicin, Pen-Strep: Penicillin-Streptomycin). Thirty-six combinations [media + serum + antibiotic] tested with three replicates per combination. [ANCOVA: df = 6, F = 0.48, p = 0.82].

**Fig 7:**
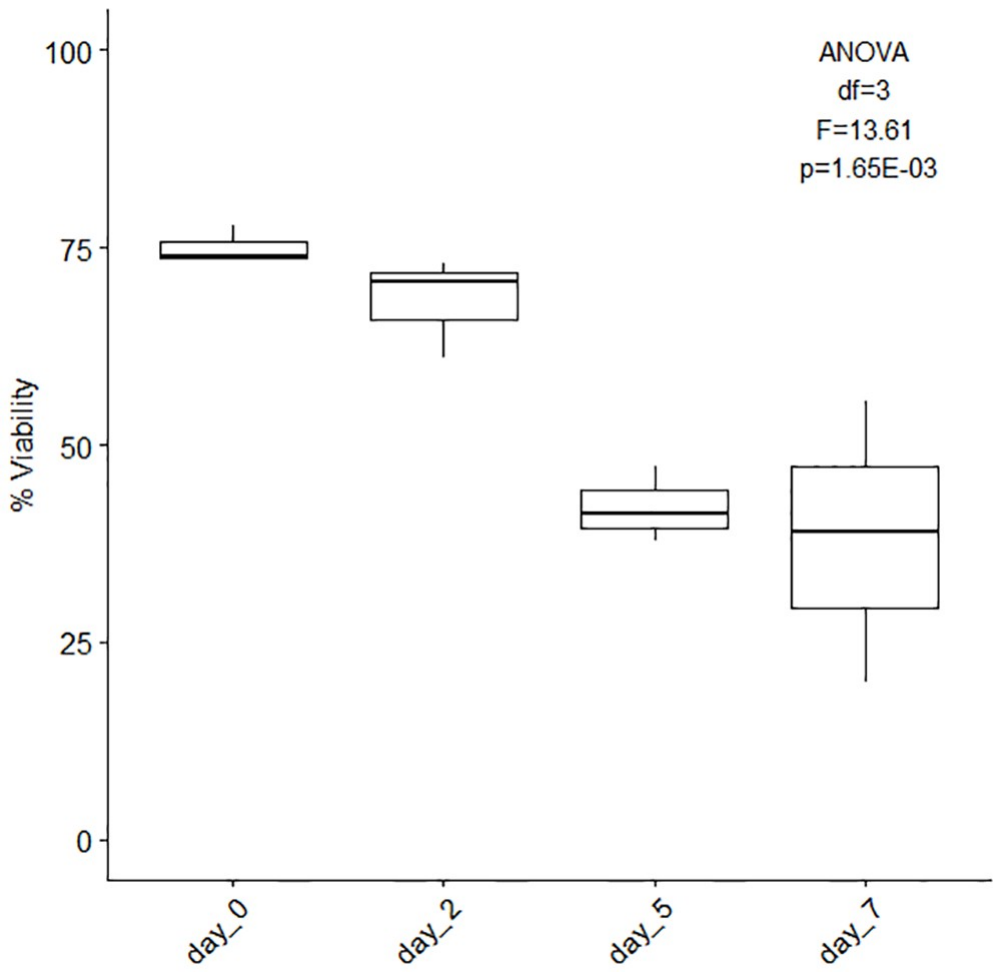
Cell viability (%) of 7-day cultures. Average overall cell viability (%, ± SE) of 7-day cultures. The cells were initially dissociated using Ca-Mg free seawater incubation for 1 hour and the culture medium used was composed of 15% DMEM, 10% FBS, 1% Penicillin-Streptomycin and 74% filtered artificial sterile seawater (replenished on days 2 and 5), n = 3. [ANOVA single factor, df = 3, F = 13.61, p = 1.65E-03].

To encourage cell proliferation and growth different cell culture media combinations were tested using Fetal Bovine Serum (FBS: 0%, 5% and 10%), Dulbecco’s Modified Eagle Medium (DMEM), Roswell Park Memorial Institute medium (RPMI), or Ham’s F12 medium, and different antibiotic combinations (Gentamicin, Antibiotic-Antimycotic, Penicillin-Streptomycin, Gentamicin + Antibiotic-Antimycotic, S2 File, S6 and S7 Tables). Systematic observation revealed the average maximum uncontaminated number of days (7 days) was reached using 10% FBS with DMEM and Penicillin Streptomycin, with media replenishment every day (Fig 6). The media combinations presenting contamination from day 1 did not contain any FBS (Fig 6, FBS: significant effect, ANCOVA df = 1, F = 48.41, p = 7.04E-10, S6 Table) and the longest lasting, contamination-free combinations were made of 5%FBS + Antibiotic-Antimycotic (with and without Gentamicin) regardless of the base medium used (DMEM, RPMI or F12). While contamination is problematic, antibiotic type did not significantly affect contamination rates (ANCOVA df = 3, F = 2.48, p = 0.06, S6 Table). Cell viability was measured on 7-day cultures (15% DMEM + 5%FBS + 1% Antibiotic-Antimycotic + 79% filtered artificial sterile seawater, media replenished on days 2 and 5) of cell dissociated using Ca-Mg free seawater incubation (1 hour) Fig 7, S8 Table. The data show a ~7, ~33 and ~37 point-decrease in percent cell viability after two, five and seven days of culture (Fig 7, S8 Table).

Cultures without FBS show the fastest rate of contamination and the FBS concentration significantly impacts contamination overall (df = 1, F = 48.41, p = 7.04E-10). Cultures with Anti-Anti and Anti-Anti+Gentamicin show the most consistent average number of days without contamination across the three different media types (6 and 5 days respectively). ANCOVA results presented in S7 Table.

### Coral cells: Type and morphology

Data quality, reproducibility and scientific rigor are fundamental to ongoing applications and future work. The minimum information guidelines require that benchmark measurements be made regularly to attest of the robustness of the protocols followed. Typical measurements of health in cell cultures are made to detect the onset of cell death or cell stress. These critical parameters include growth rate (doubling time), morphology (size, shape, *in vitro* and *in hospite*), concentration, and viability or apoptosis. However, benchmarks indicating the success of coral cell cultures have yet to be established due to the difficulties linked to keeping coral cell cultures over extended periods of time. Nevertheless, certain parameters have been identified in published works and summarized in Table 1. These parameters mainly refer to cell morphology in the case of nematocysts, mucocytes and desmocytes nevertheless calicoblastic cells and symbiotic cells, because their particular functions have attracted more scientific attention, can also be characterized in relation to other parameters such as the type of calcium carbonate (CaCO_3_) precipitated, or fluorescence and density for symbiotic cells.

**Table 1:** Benchmark measurements according to cell type, *in hospite* and *in vitro* for coral cell line cultures.

Cell Type	Indicator	*In Hospite* Expected Range	*In Vitro* Expected Range	Reference
**Ectodermal cells (oral tissue)**				
**Nematocyst**	**Cell shape**	*Acropora hyacinthus*: rounded rectangular shape; *Galaxea fascicularis*, spirocyst type 1: thin and elongated; *Galaxea fascicularis*, spirocyst type 2: thicker than type 1; *Galaxea fascicularis*, spirocyst type 3: rounded oval shape;	*Pocillopora damicornis*: spindle shape; *Galaxea fascicularis*, spirocyst type 1: thick spindle-shaped capsule with sparsely (single coil) barbed shaft; *Galaxea fascicularis*, spirocyst type 2: thin spindle-shaped capsule with densely barbed (helix) shaft; *Galaxea fascicularis*, spirocyst type 3: oval-shaped with one sharper end; *Pocillopora actua*: mastigophore and trichous haploneme: oblong; spirocyst: oblong with visible coil inside;	[97,104–109] This study
	**Cell size**	*Acropora hyacinthus*: ~5μm x 20μm; *Galaxea fascicularis*, spirocyst type 1: ~100μm x 800μm; *Galaxea fascicularis*, spirocyst type 2: ~100μm x 800μm; *Galaxea fascicularis*, spirocyst type 3: ~100μm x 200μm;	*Pocillopora damicornis*: 7.5μm by 30μm; *Galaxea fascicularis*, spirocyst type 1: capsule 10μm by 30μm, shaft 15μm; *Galaxea fascicularis*, spirocyst type 2: capsule 3μm by 30μm, shaft 20μm; *Galaxea fascicularis*, spirocyst type 3: capsule 6.5 by 13μm; *Pocillopora acuta* mastigophore ~130μm x 40 μm with 1.5mm rod; trichous haploneme ~250μm x 30μm with 260μm rod; spirocyst: 120μm;	[97,105–109] This study
**Mucocyte**	**Cell shape**	*Acropora hyacinthus*: thin and elongated, rectangular; *Galaxea fascicularis*: thick and elongated, densely packed along the ectoderm of “sweeper tentacles” but rare along the “catch tentacles”; *Coelastrea aspera*: oval shaped; *Montastraea annularis*: rounded rectangular shape; *Galaxea fascicularis*: elongated drop-shaped; *Goniastrea aspera*: elongated drop-shaped;	Unreported	[97,105–109]
	**Cell size**	*Galaxea fascicularis*: ~150μm x 500μm; *Galaxea fascicularis*: ~7μm x 35μm; *Coelastrea aspera*: ~9μm x 11μm; *Acropora hyacinthus*: ~10μm x 20μm; *Montastraea annularis*: ~15μm x 30μm; *Goniastrea aspera*: ~12.5μm x 40μm;	Unreported	[97,105–109]
**Calicoblastic Cells**				
	**Cell shape / description**	“Long-thin-tall” to “thick and cup-like” Note: flat calicoblastic cells tend to manifest low calcifying activity whereas cup-like cells manifest the opposite	Rounded	[110,111]
	**Cell size**	*Stylophora pistillata*: < 6μm; *Stylophora pistillata*: 4–30μm; *Stylophora pistillata*: 5–10μm;	*Stylophora pistillata*: 5–6μm	[94,110,112]
	**CaCO**_**3**_ **crystal precipitation**	*Stylophora pistillata*: 6.5–12.5μm rod-shaped or spherical-looking; *Stylophora pistillata*: 2μm width, rod-shaped, growing in length; *Stylophora pistillata*: 1–10μm fiber bundles (0.5–1μm fibers); *Stylophora pistillata*: 4–10μm spherulite-like nanogranules, later becoming 10–35μm dumbbells; *Stylophora pistillata*: 2.5–5μm spherulite-like nanogranules; *Pocillopora acuta*: 1–5μm rod-shaped, aggregated into dumbbells 15–30μm in length;	*Stylophora pistillata*: 10μm	[90,93,94,113,114]
**Symbiotic Cells**				
	**Symbiont cell size**	*D*. *glynnii*: length 9.52 ±0.31(SD), width 8.43 ±0.39 (SD); *C*. *goreaui*: length 10.80 ±0.88 (SD), width 10.40 ±0.83 (SD)	*D*. *glynnii* and *Cladocopium* sp. are not culturable, *in vitro* values are derived from culturable strains from the same genus *Durusdinium* cell volume: 246–1124 μm^3^. *Cladocopium* cell volume: 220–1586 μm^3^	[26,65,115–117]
	**Symbiont cell density**	3.5 x 10^5^–3 x 10^6^ cells/cm^2^	Varies with growth phases	[118–120]
	**Symbiont PSII photochemical efficiency (F**_**v**_**/F**_**m**_**)**	~0.5–0.7	*Durusdinium spp*. 0.37–0.52 *Cladocopium spp*. 0.41–0.53	[67,116,117,121,122]
**Other**				
**Desmocyte**	**Cell shape**	*Mycetophyllia reesi*: rounded	Unreported	[123]
	**Cell size**	*Mycetophyllia reesi*: 15μm x 20μm	Unreported	[123]
**Amoebocyte**	**Cell shape** **Cell size**	Unreported	*Pocillopora acuta*: roughly rounded with granular surface; *Pocillopora acuta*: 20–30μm	This study This study

Not all cell types are presented here because of the limited data available. Tresguerres et al. [124] present nine cells types: epitheliomuscular cells, nematocysts, ciliated support cells, calicoblastic cells, symbiotic cells, desmocytes, neurons, mucocytes and pigment cells, whereas Rosental et al. [75] identify 12 cell populations in scleractinian coral *Pocillopora acuta* through the use of FACS, and Hu et al. [76] indicate 16 cell populations in soft coral *Xenia* through a combination of FACS and single cell RNASeq. Table 1 presents several key cell types and their *in hospite* and *in vitro* characteristics alongside our measurements to serve as a reference for ongoing coral cell culture research.

**Fig 8:**
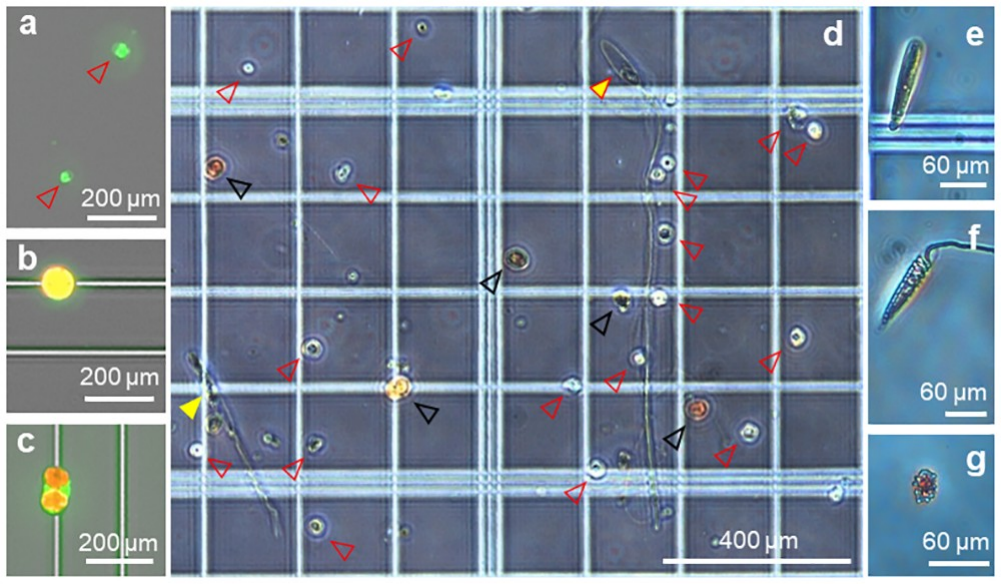
Coral cell variety (microscope photographs). Different cell types observed after dissociation from *P*. *acuta* nubbbins, coral host cells of different sizes (a, red arrow heads), single Symbiodinaceae cell (b), two symbiotic cells encapsulated inside a coral host cell (c). (d) unsorted cells composed of miscellaneous coral host cells (red arrow heads), symbiotic Symbiodinaceae (black arrow heads), deployed trichous haploneme nematocyst (yellow arrow head) and deployed mastigophore nematocyst (yellow arrow head with red outline), closed capsule of trichous haploneme nematocyst (e), spirocyst nematocyst (f) and amoebocyte-like cell (g). Photographs (a)–(c) were taken using a Cytation3 imaging plate reader with GFP and Texas Red filters. Photographs (d–g) were taken under a compound microscope (no staining).

Cell dissociation allowed us to observe different types of cells: symbiotic cells (singletons or doublets within a coral cell or free-living), nematocysts, undetermined host cells and amoebocytes (Fig 8). Host coral cells measured ~10 μm x 13 μm and symbiotic cells measured ~40–50 μm in diameter (Fig 8b–8d). Four different types of nematocyst were observed: mastigophore nematocyst ~130 μm x 40 μm with 1.5mm rod (Fig 8d), trichous haploneme nematocyst ~250 μm x 30 μm with 260 μm rod (Fig 8d), trichous haploneme nematocyst capsule ~120–150 μm in length (Fig 8d) and spirocyst ~120 μm in length (Fig 8f). Amoebocyte-like cells were also visible ~20–30 μm x 10 μm (Fig 8f).

Growing evidence suggests marine invertebrate performance cannot be characterized using a single parameter (i.e. growth rate or morphology or concentration or viability), but rather an ensemble of interdependent factors [122,125,126]. While Symbiodiniaceae density and activity, and calcium carbonate precipitation by calicoblastic cells can be measured, coral cellular activity of neurons, nematocysts and amoebocytes have not yet been investigated. Moreover, the complexity of coral microbiomes and the associated interdependencies [78] have not been sufficiently characterized to make any overarching assumptions of how the microbiome influences coral cell health in culture compared to *in hospite* interactions.

### Evaluation of methods

Testing various cell culture methods enables their critical comparison and advantages and disadvantages for each method tested are summarized in Table 2. Exact protocols applied will be dependent on the optimal experimental designs for the hypotheses being tested. Nevertheless, quantifying variability and identifying the sources of this variability is fundamental for comparative assessment [e.g. 127], see variability in Table 1.

**Table 2:** Summarized advantages and disadvantages of each method tested for coral cell culture.

Method used	Advantages	Disadvantages
**Cell dissociation**		
By simple washing By mechanical scraping with razor blade or scalpel	Easy; Adapted to species with large polyps and/or large tentacles;	Not very effective; Time consuming; yielding ~50%; not adapted to species with small polyps and/or small tentacles;
By paint brush/toothbrush	Relatively easy to perform regardless of the polyp size;	Can be rough for cell membranes and lead to contamination from the mucus layer; time-consuming;
By Ca^2+^-, Mg^2+^-free seawater	Simple incubation method;	Effect on desmocytes unknown; mix of single cells and incompletely dissociated tissue fragments detach from the skeleton;
**Cell digestion**		
Enzyme digestion: trypsin	Simple incubation method; high yield; converts proteins to peptides; can promote cell aggregation;	Effect on desmocytes unknown; no standard concentrations or incubation time available; cell clusters; can damage cell surface proteins and their subsequent adhesion capacity; chelating agent necessary to ensure effectiveness (e.g. EDTA);
Enzyme digestion: liberase	Simple incubation method; high yield especially for algae cells;	Effect on desmocytes unknown; no standard concentrations or incubation time available; can affect cell adhesion/aggregation capacity, often presented as a blend of various enzymes;
**Cell sorting**		
Percoll density gradient	Only small doses of Percoll needed each time;	Not cost-effective; density gradients are not always marked enough; protocol must be optimized accordingly to the density characteristics of targeted cells and multiple successive Percoll step gradients needed for full separation;
FACS	Staining process is simple; a lot of data is generated (cell counts per population and relative cell sizes);	Multiple dyes can be costly; partially based on ROS concentration; multiple passes needed for full separation; still new method applied to coral cells;
**Growth medium**	Methods established with other living organisms; many products available to purchase;	Does not correspond exactly to coral cell needs; must be combined with additives for optimization;
DMEM		
RPMI		
F12		
FBS		
**Antibiotic treatment**		
Pen-Strep (Penicillin and Streptomycin)	Method established with other living organisms; many products available to purchase;	Little effect on the bacterial and viral population observed;
Anti-Anti (Penicillin, Streptomycin, Amphotericin B);	Method established with other living organisms; many products available to purchase; More effective than Pen-Strep alone;	
Gentamicin	Method established with other living organisms; many products available to purchase;	Most efficient when combined with other antibiotics that target DNA synthesis;
**Cell attachment**		
Untreated glass or plastic	Simple;	~40% of cells lost;
Collagen coating (glass or plastic)	Can be tested with macromolecules other than collagen;	Might need to be tailored to different cell types;
**Cell visualization for counting**		
Trypan Blue	No incubation necessary;	Lethal stain; reacts with salts and creates clusters;

## Discussion

Advances in coral aquaculture in recent decades, have provided fine-scale control over many key environmental factors to investigate the impact of change on multiple coral species raised *ex situ* in aquaria, but survival of isolated coral cells and obligate symbiont cells is still very limited [78,128]. Furthermore, despite the growing number of studies on coral bleaching (e.g., [129], and reference therein, diseases [e.g. 130–132, and references therein] and toxicity [e.g. 133–135], a concrete list of coral “health” indicators has yet to be established [68,136,137]. Several main indicators of coral performance that are commonly applied at the colony or fragment level include: polyp activity [i.e. extension, feeding, 117,138], tissue thickness, [118,138,139], biomass [140], symbiosis using symbiont density and green fluorescent protein intensity [141–143], microbiome distribution and composition [144–149] and skeletal growth [150,151]). With the advent of microsensors and nano scale assessment, more subtle metrics have also been identified over the past decade, such as ciliary beating [152] and the physiochemical equilibrium of the skeleton calcification mechanism [153,154]. These indicators mostly relate to polyp- and colony-level changes since most studies utilize primarily coral fragments for research, furthermore, some of these indicators have non-negligible limitations. Coral growth, for example, has long been considered as an important performance metric so, traditionally, the rate of linear extension was considered a measure of coral fitness because coral reproduction is based on size [155]. However, growth has also been demonstrated as highly variable according to parameters such as seawater temperature [156] and pH [155], and recent studies have identified limitations of tracking growth as a primary indicator of performance [125,157], with multiple metrics likely a necessity.

Through model systems approaches we can work towards identifying and standardizing the different metrics necessary to better define coral health and to make scientific findings more consistently comparable across scales and species. Coral cell lines, or at least long-lived cultures, represent a key method for applying the model systems toolkit and can catalyze research advances in coral biology. Testing the protocols developed by different research groups allowed us to highlight simple and effective cell dissociation methods (Ca^2+^-Mg^2+^ free seawater incubation for 1 hour) plus potential challenges with common approaches (e.g. trypan blue) to simplify and widen the range of applications of coral cell cultures. This work has also allowed us to describe three different types of nematocysts and amoebocyte-like cells in *P*. *acuta*. Together our findings represent an advance towards a better understanding of coral cell biology. As we continue the optimization of coral cell culture, certain improvements remain to be made, namely tuning the culture media mixture to maximize cell survival and promote proliferation, as well as identifying the functions of the different cell populations.

### Consolidating methods

#### Dissociation

The preparation of cell suspensions is a crucial factor for any successful cell culture-based experiment. The ideal method should isolate the desired cells from tissue samples while avoiding cell aggregation, preserving cell viability and cell surface markers for sorting and other experiments, e.g. immunophenotyping [158].

The first step, cell dissociation, can be performed using four different popular methods: mechanical scraping (using a scalpel), brushing (with soft paint brush vs. toothbrush), Ca^2+^-, Mg^2+^-free seawater incubation or enzymatic digestion. The first two methods are prone to person-to-person variability, instrument characteristics and possible species variations (e.g. large vs small polyps, S1 Table. variability). Although all methods are effective, questions remain concerning cellular integrity after disruption of cell-to-matrix and cell-to-cell interactions. The vital stain used to count cells in the present study (trypan blue, recommended in the literature) reacted to seawater, creating blue clusters impairing visualization of staining efficacy. Enzymatic digestion using collagenase, has, for example, the potential to stimulate reprogramming and transdifferentiation of cell types [e.g. *Podocoryne carnea*, jellyfish, 128,159,160] therefore, functional characteristics specific to different cell types and phenotypic stability need to be followed closely when enzyme digestion is involved [128,159]. Applied to coral cells, trypsin-EDTA treatment was reported most effective [high yield, 99,128] compared to collagenase and pronase treatment, although trypsin can damage cell surfaces thereby impacting future adhesive capacities similarly to liberase blends made of pronase and collagenase [97,101]. Conflicting reports regarding cell surface damage using trypsin and liberase [86,97,101,128] can be attributed to the blend used and how rarely the extract enzymatic reagent blend is specified by the manufacturer. Our findings suggest combining mechanical dissociation and enzyme digestion (trypsin) can yield double the number of cells (Fig 4) with only a slight decrease in viability (-2.9%). While cell reaggregation capacity is not cardinal, cell adhesion is, especially for long term cell cultures. Mechanical scraping and Ca^2+^-, Mg^2+^-free seawater incubation also disrupt the cells responsible for the attachment of the soft tissue to the coral skeleton, desmocytes. This attachment disruption phenomenon and its cascading effects have yet to be fully investigated [128]. Studies focusing on desmocytes should carefully assess cell dissociation methods with the goal of minimal disruption in mind. Coral cell dissociation is a more delicate process than expected and considering the drawbacks of each approach is important to designing protocols adapted to the nature of each study.

Domart-Coulon et al. [83] report a yield of 0.5–1 x 10^6^ cells from a 0.3–0.5 mm long coral nubbin after 3 h incubation in Ca^2+^-, Mg^2+^-free seawater. Replicating this method, we were able to dissociate 5.9–7.6 x 10^6^ cells (S1 Table) from a 0.5–0.9 mm long coral nubbins, which aligns with the previously reported findings. Ca^2+^-, Mg^2+^-free seawater incubation is an effective, easy and cost-efficient method with high cell yields. Results show that optimum incubation time is 1 hour. While extended incubation periods could help dissociate different types of cells, the cell yield found after 1 hour incubation is sufficient for cell cultures. Further work should attempt to separate and identify the different populations dissociated at different time points during Ca^2+^-, Mg^2+^-free seawater incubation. This could also be done when comparing simple mechanical dissociation with mechanical dissociation and enzyme digestion).

#### Cell separation

The next step consists in separating the cell mixture into its individual constituent cell types to achieve monocultures of different coral cell populations. Density gradient centrifugation is reported to easily separate coral cells from contaminating bacterial cells with high-purity and high-yield [102]. Nevertheless, when tested, this method did not present the clear density gradient expected and the reagent cost significantly outweighed the effectiveness. While FACS method is more effective at separating coral host cells from symbiont cells, further separation partially relies on the differential concentration of reactive oxygen species (ROS) among different cell types [75,95]. Although innovative and effective, this method relies on a very active biological phenomenon, ROS production linked to oxidative stress, that is influenced by the biology of the cells and by every processing step prior to analysis with the FACS machine. To date, the production of ROS is still not well characterized in coral cells and variability in ROS concentration and type might lead to mixed populations. Label-free cell separation using inertial microfluidics devices is a promising method recently reviewed by Gou et al. [161]. The recent advances in the field of microfluidics and the characterization of force and flow now allow us to circulate, sort and enrich different cells, e.g. tumor cells, exosomes, DNA and other biological materials, [161] and references therein. Simple devices can be engineered to sort the cells but, like the density gradient centrifugation and FACS methods, the system needs to be tailored to the different types of cells targeted, and full separation might involve multiple passes through the inertial microfluidics device [161]. Methods of cell separation such as the combination of affinity ligands to microfluidic devices, magnetic activated cell sorting, cell affinity chromatography or expanded bed chromatography (reviewed in 162) should also be investigated in relation to coral cells to overcome issues related to *in vitro* cell morphology, i.e. different coral cell types tend to be very similar. Cell separation technology has progressed steadily despite lingering challenges, including meeting basic characteristics of rapidity, efficacy and affordability while maintaining high yield, purity and cellular functionality [162]. Bacon et al. [162] single out membrane-based separation combined to specific biorecognition moieties as a method that ensures high yield, purity and cellular functionality while allowing high throughput, reduced processing time and maintaining high viability.

Cell population separation is an important step that needs to be complemented by the identification of these populations. Rosental et al. [75] and Snyder et al. [95] were successful at separating symbiotic populations from asymbiotic populations but further identification or cell typing is needed. Identification could be undertaken by monitoring different parameters, e.g. granularity, size and shape, and it was observed in this study that cells become more rounded with increasing time in culture. FACS could further help the identification through forward and side scatter but no population specific ranges have yet been determined.

#### Proliferation

Once the coral tissue is successfully detached from the skeleton and the cells sorted according to their functionality, finding the optimal growth medium mixture is key to cell survival [163]. Culture media selection rests on the assumption that the closer media composition is to the metabolic requirements of the organism, the more successful the cell culture will be. Growth media should be comprised of amino acids, nucleic acids, vitamins, carbohydrates (glucose, galactose, maltose, fructose), inorganic salts (Ca^2+^, Mg^2+^, Sr^2+^), buffering agents for pH and osmolarity, and (animal) serum, which contains lipids, proteins (albumin, transferrin, aprotinin, fetuin, fibronectin, collagen), growth factors, attachment factors, hormones. The media cocktails tested here on mixed coral cell populations, showed that FBS is a key ingredient since cultures with 0% FBS were nearly all contaminated after 1 day. Contamination occurred in every culture regardless of the base medium used (DMEM, RPMI or F12, see S9 Table for composition) but the added antibiotic-antimycotic and Penicillin Streptomycin treatments seemed to inhibit contamination the longest (Fig 6, average maximum number of days without contamination: 7 days; maximum number of days without contamination: 10 days), combined to a 10–15 min aerated iodine dip of the nubbins before cell dissociation (Reef Dip^™^ Coral disinfectant, Seachem). A better control of the microbial population needs to be achieved to design a better adapted culture medium for long term cultures. Furthermore, a “one-size-fits-all” approach to culture medium composition (S9 Table) may not yield consistent success rates among cell types because some coral cells harbor endosymbiotic dinoflagellates (Symbiodiniaceae). The different nutrient needs associated with each organism (i.e., Symbiodiniaceae and coral, [164–168]) need to be reflected in the different media blends to generate optimal yields (e.g. growth, isolation success rates, adhesion) and the exchanges between coral host and symbiont need to be carefully considered (e.g. amino acid synthesis, carbon and nitrogen source/sink [169]. The Currently available artificial growth media are poorly adapted to the diversity of coral cells as survival has not been achieved beyond one month [96].

Coral colonies have diverse microbiomes [e.g. 144,170–173] and microbial contamination of coral cell culture is problematic. Thorough initial sample rinsing can reduce the initial concentration of microorganisms, which often reside in the coral mucus, and the addition of antibiotics can inhibit the growth of bacterial and viral communities associated with coral cells. Anti-bacterial, -viral cocktails for coral cell cultures are poorly described and rarely justified through isolation and identification of problematic bacteria or viruses. Researchers have used 1% antibiotic cocktail (streptomycin–gentamycin, 1:1 ratio) to extract cells from soft coral *Sinularia flexibilis* [101]. Lecointe et al. [97] used seawater supplemented with (v:v) 3% antibiotics-antimycotics solution (AB-AM, Gibco/Life Technologies, Carlsbad, CA, USA) with final concentration of Amphotericin B <0.3%, Penicillin 1.5–4.5%, Streptomycin 1.5–4.5% for coral cell isolation from *P*. *damicornis* nubbins [97]. The antibiotic cocktails tested here showed that gentamicin alone was not sufficient to control the bacterial/viral population. The antibiotic-antimycotic treatment was the most consistent at controlling contamination compared to penicillin-streptomycin which varied with FBS concentration. Without a more in depth understanding of the bacterial and viral populations contaminating the coral cell cultures, it remains unclear which antibiotic combination is the best suited to prevent contamination over the longer term.

#### Cell attachment

Cell attachment is important to long-term culture and proliferation. Multiple substrates can be tailored to promote cell adhesion: tissue culture treated plastic, Primaria, and glass, with or without the use of surface coatings (e.g. collagen, poly-L-lysine, RGD-[e[tide). Amongst these techniques, the latter is the most open to innovations with the testing of different surface coating in accordance with biomimicking properties of the extracellular matrix (ECM) and the mesoglea, similarly to growth media engineering. As the ECM is the fundamental attachment medium for cnidarian cells, testing a number of its components could lead to very efficient surface coating to promote cell adhesion. Collagen forms a unique gel-like fibrillary layer in corals and is mainly composed of repeating glycine and hydroxyproline. Collagen is thought to be the main macromolecule responsible for cell bonds and, *a fortiori*, cell attachment. Contrary to expectations, collagen coated glass and plastic were less successful at promoting cell attachment compared to their uncoated counterparts. Other molecules, such as fibronectin, laminin, chitosan and the polysaccharide HSPG (heparin sulfate proteoglycan) could potentially promote adhesion [128,174] better than the collagen tested. Furthermore, testing different sources and types of collagen might help narrow down the essential ingredients for cell adhesion. Investigation of the composition of the mesoglea and the skeletal organic matrix (SOM) could lead to more testable molecules, but the SOM is notoriously difficult to isolate without losing the soluble fraction, or without residual tissue contamination [e.g. 85 and associated letters], thus SOM investigations require caution.

### Benchmark measurements

Complete and accurate reporting of relevant information is essential to allow methods and protocol reproduction, and enable community adoption, meta-data analyses, modelling, systematic comparison and standard refinement [175]. Minimum information standards have been presented in an attempt to reduce animal testing, reduce financial waste and improve bioscience research reporting [e.g. 180,181]. Such guidelines can be applied here but the reporting of results and observations needs to be adapted to the complexity of reef-building corals. Considering the limited information available on coral cells even basic information, such as shape and size, need to be included as benchmark measurements along with Symbiodiniaceae activity and cell survival throughout culture and experimental period.

#### Cytotoxicity

Cell viability must be established for successful cell line generation regardless of cell origin. To this effect cytotoxicity assays need to be reliable, straightforward and relatively rapid to react. Methods for assessing cell membrane integrity include dye exclusion assays [e.g. trypan blue, 73,95], Evan’s blue [176,177], propidium iodide [75], SYTOX green [85] and enzyme release assays (e.g. lactate dehydrogenase, LDH, [178]. Intracellular enzymatic activity has also been used as indicators of cell viability, such as measurement of esterase activity using fluorescein diacetate [27] and mineralization activity in calcifying cells using the alkaline phosphatase assay [83]. Assays measuring metabolic activity either directly (e.g. ATP bioluminescence) or indirectly via dye conversion (e.g. MTT (3-[4,5-dimethylthiazol-2-yl]-2,5-dipheny tetrazolium bromide assay, 100) have also been used. Additionally, metabolic activity can be characterized through measurements of mitochondrial properties such as membrane potential (JC9 dye) and density (MAO dye, 100). The method used during our testing was Trypan blue staining. It is a simple and well-established method that stains dead cell membranes and tissues blue. Unfortunately, trypan blue reacts with seawater and proteins (from culture media) creating clusters that make observations difficult. Furthermore, trypan blue is a lethal stain which cannot be used to assess cell viability during culture. An approach using non-lethal vital stains should be preferred. Neutral red could be an alternative depending on the time needed for the cells to take up the stain (incubation). Neutral red staining protocols recommend a 2 hour incubation at culture temperature [179], 25°C in the case of corals, but this considerably extends the handling time, which could affect results. Further testing will determine whether neutral red is a suitable vital stain for coral cell viability measurements. DAPI (4′,6-diamidino-2-phenylindole) is also commonly used to stain dead cells. Unfortunately, DAPI stains dead cells with compromised membrane integrity (necrosis) leaving intact dead cells (apoptosis) unstained. Investigations into fluorescent stains more adapted to coral cells should be undertaken, especially considering the strong autofluorescence of both host coral cells and symbiotic algae cells.

When cell death is measured, additional studies to elucidate potential mechanisms of toxicity are conducted, which can include oxidative stress experiments to determine any imbalance in reactive oxygen or nitrogen species generation and genotoxicity experiments to assess the extent of DNA damage. A majority of coral cell studies have only focused on establishing cell cultures which remain viable beyond a few days. Therefore, additional research is needed to assess sublethal cellular level changes due to culture conditions and/or physico-chemical exposures.

It should be noted that in relation to culture, cell survival can be overshadowed by potential bacterial activity even with small, non-critical, levels of contamination (i.e. culture contamination is not necessarily synonymous with cell death). Considering the fragile balance corals have with their microbiome, low contamination levels may not be problematic to the coral cell culture itself but could interfere with cytotoxicity assessments.

#### Cell morphology and functionality

The cell dissociation allowed us to differentiate Symbiodiniaceae from other coral cells, along with multiple different types of nematocysts and amoebocyte-like cells. While nematocysts have been well studied in anemones, little data is available on coral nematocysts. Across Cnidaria, ~30 different types of nematocysts exist with high diversity amongst Medusozoa [180]. The diversity and complexity of nematocysts increased through evolution, from Anthozoa to Medusozoa and species are reported to each have between 2–6 different types [180]. Nematocysts are classified according to their shape. Reef building corals present three types of nematocysts: trichous haplonemes, spirocysts and mastigophores [180]. The nematocysts observed in *P*. *acuta* follow this rule (Fig 8e) showing a closed trichous haploneme capsule, a deployed mastigophore and a deployed trichous haploneme (Fig 8d), and a spirocyst (Fig 8f). The function of nematocysts is to capture prey and defend against predation. Kass-Simon & Scappaticci [181] voice the fascination surrounding the potential for nematocysts to act independently, without neuronal intervention. Certain nematocysts in Hydra are reported to contribute to polyp locomotion [181] but this has not been investigated in coral polyps to date. It is also unknown how nematocysts respond to physiological stress (e.g. suppressed activity in bleached corals and potentially heightened coral starvation, potential involvement in polyp bailout).

Amoebocytes (Fig 8g) are part of the inflammatory response related to injury. They are the putative immunocytes of the anthozoans [182] and poorly understood in scleractinian corals. Cultures of amoebocytes could lead to considerable breakthrough in the field of wound healing and tissue regeneration. This is particularly relevant today, with thermal stress compromising immune responses [183] and the increased frequency of marine heat-waves. Indeed, yellow band disease combined with thermal stress exhausted immune defenses of coral *Montastraea faveolata*, thereby letting pathogens colonize healthy tissue and precipitating colony death [183]. The complexity of the holobiont added to the combined effects of disease and thermal stress makes for an intricate system to understand. Amoebocytes culture could potentially help deconvolute the interconnections and identify whether colony death was due to the suppression of certain immune factors or the increased pathogen virulence.

### Other important considerations

#### Symbiodiniaceae

The physiological upkeep of Symbiodiniaceae is paramount to the maintenance of coral-dinoflagellate mutualisms and therefore an important metric to gauge the success of cell culturing efforts. Tracking Symbiodiniaceae cell density is important for determining whether symbiosis has been re-established and for ensuring the further growth of cultures and can be monitored via microscopy. The use of photosystem II photochemical efficiency (F_v_/F_m_) serves as a benchmark for efficiency of photochemistry and allows for the comparison of cell culture health to a wide range of coral physiology and bleaching studies. Additionally, these indicators are important when gauging the success of culturing aposymbiotic cell lines because they can serve as quick indicators of contamination.

#### Cryopreservation

Cryopreservation of cells is advantageous for basic research as it provides a means to conserve cells or tissue for later use, allowing for experimentation without the need to collect fresh samples each time. Cryoprotectants, such as dimethyl sulfoxide (DMSO), are often employed to prevent ice formation, which can damage and reduce survival of cells. Feuillassier et al. [184] compared the effectiveness of ethylene glycol, DMSO, methanol and glycerol on preserving *Pocillopora damicornis* tissue balls and, based on tissue ball regression DMSO, ethylene glycol, and glycerol were determined to have the least toxicity.

#### Imaging

Imaging is a versatile method to explore structures and fundamental properties of coral reefs in the scales of millimeters to meters. At high-resolution, microscopy-based studies of corals lead to the clear interpretation of physical and biological processes governing coral health and proliferation. Tissue or cellular level coral physiology have been explored using advanced high-resolution microscopy methods, e.g. bright field microscopy, phase-contrast microscopy, differential interference-contrast microscopy [102,185]. Typical experimental examples are the observations of calcareous skeleton, decalcified coral tissues [102,185,186] and the physiological and nutritional status of their symbiotic dinoflagellates [187–190]. These successful attempts provide a great deal of information at the microscopic level, i.e. cellular and subcellular structures. However, the interaction of calcifying cells with the calcareous skeleton cannot be observed once the tissue is separated from the skeleton, and traditional tissue sections rely on fixatives that can create artifacts. Three-dimensional interactions between coral cells, symbiotic cells and the skeleton have therefore been limited. Moreover, dynamic processes *in vivo* as a function of time cannot be revealed at the tissue and cellular levels using these methods. In order to solve this problem, some studies put forward the cell or tissue cultures as the miniaturized model, which can be studied *in vitro* in real time. Although this method provides an alternative to study the physiological processes involved in symbiosis and calcification, the generated cell or tissue cultures extracted from coral tissue will lose the tissue structures and organizations [83,85]. Besides, cells or tissues in cultures are in a state of declining health, which may not reflect the whole physiological and metabolic processes of corals [78,85,93].

Fluorescence microscopy opens up an avenue to use the autofluorescence of coral tissue as an indicator of coral performance [118,191,192]. This non-invasive method can provide a way to obtain the interaction of coral tissues as well as the dynamic processes *in vivo* in real time. For example, live imaging Confocal Laser Scanning Microscopy (CLSM) or Confocal Raman Microscopy (CRM) have allowed us to examine calcification [90,92], intracellular pH [193–196], tissue thickness and innate Symbiodinaceae autofluorescence [118,120,197], although these investigations are limited to relatively short duration. CLSM has also been used to quantify disease-induced changes in coral fluorescence associated with tissue loss diseases in *Montipora capitata* [191].

## Conclusions

The work presented here establishes a framework for the development of immortal coral cell cultures and the application of model systems approaches to reef-building corals as non-model systems. The thorough comparison of coral cell dissociation methods highlighted different cell yields but preserving cell integrity and function is essential, as are ways to successfully assess both parameters. The various culture media combination tested show the composition needs to be tailored to extend cultures beyond one week and suppress contamination for longer periods. Furthermore, if the model systems approach is to be further applied to coral cell cultures, moving away from mammalian-based media supplements (e.g. FBS and basal media) is the next step. To this end, the cell-specific requirements of each coral cell type need to be investigated and matched with the right supplements and concentrations. The cell attachment tests performed in this study reveal typical surface coating for mammalian cell cultures do not promote coral cell attachment and different coating should be tested. Finally, coral cell identification needs to be more advanced before the field can move towards establishing rigorous and streamlined cell-based culture methods for a variety of cell types mirroring model systems’, e.g. in silico cell identification, advanced cell separation, immortal monoculture cell lines. Only then we will be able to fully understand the molecular mechanism associated with coral cell biology and functions.

## Supporting information

S1 Table Methods tested for different steps of coral cell culture. Parameters: Yield, cell viability, reproducibility of method, effectiveness, incubation time.
(DOCX)

S2 Table Cell dissociation method comparison data. Algae cell and coral cell yields as a factor of dissociation method (washing, soft/hard brushing, mechanical scraping, or calcium-magnesium-free seawater incubation for 1 to 24 hours) and enzyme digestion (Trypsin or Liberase).
(DOCX)

S3 Table Overall cell viability immediately after dissociation, as a factor of dissociation method: Data. Percent viability of coral cells dissociated using different methods (soft/hard brushing, mechanical scraping, or calcium-magnesium-free seawater incubation for 1 to 24 hours, and enzyme digestion). Each method was replicated at least three times.
(DOCX)

S4 Table Cell dissociation method combination efficacy data. Algae cell and coral host cell yields as a factor of the origin of cell counted: Initial cell dissociation performed by scraping (n = 18), remaining cells (on the skeleton) were dissociated using trypsin incubation for 1 hour (n = 9).
(DOCX)

S5 Table Cell attachment experiment data. Four substrates were tested for coral cell attachment: Tissue culture treated plastic (TCT), collagen coated TCT, glass and collagen coated glass. Cells were counted and the data is presented below (counts and percentages).
(DOCX)

S6 Table Culture contamination rate data. Average contamination free cell culture duration (days ± SE) as a factor of media type (F12, RPMI, DMEM), serum (0%, 5%, 10%) and antibiotic (Gentamicin, Anti-Anti: Antibiotic-Antimycotic, Anti-Anti + Gentamicin, Pen-Strep: Penicillin-Streptomycin). Thirty-six combinations [media + serum + antibiotic] tested with three replicates per combination.
(DOCX)

S7 Table Cell culture contamination variation ANCOVA results. FBS: 0%, 5%, 10%. MEDIA: F12, RPMI, DMEM. ANTIBIOTIC: Gentamicin, Antibiotic-Antimycotic, Antibiotic-Antimycotic + Gentamicin, Penicillin-Streptomycin.
(DOCX)

S8 Table Cell viability (%) after 7 days culture: Data. Percent viability of coral cells dissociated using calcium-magnesium-free seawater incubation for 1 hour and grown for 7 days in growth media (15% DMEM + 10% FBS + 1% Penicillin-Streptomycin + 74% filtered artificial sterile seawater, media replenished on days 2 and 5, n = 3).
(DOCX)

S9 Table Composition of different growth media used in this study. (DOCX)

S1 File *Pocillopora acuta* sequence and sequencing protocol. DNA sequence extracted from corals used for this study and link to associated protocol repository.
(DOCX)

S2 File Cell dissociation protocol and protocol repository. Protocols used for coral cell dissociation, attachment, culture, count and separation.
(DOCX)
