# Supplementary material for: Applying model approaches in non-model systems: A review and case study on coral cell culture
Source: PLoS One. 2021 Apr 8;16(4):e0248953. doi: 10.1371/journal.pone.0248953 (PMC8031391; doi:10.1371/journal.pone.0248953)
Supplement: S1 Table — Parameters: Yield, cell viability, reproducibility of method, effectiveness, incubation time. (DOCX) [file pone.0248953.s001.docx]

**S.2. Table**. **Methods tested for different steps of coral cell culture**. Parameters: yield, cell viability, reproducibility of method, effectiveness, incubation time.

| **Method used** | **Parameter** | | **Parameter** | |
| --- | --- | --- | --- | --- |
| **Cell isolation** | **Mean yield ± SE (cell/cm^2^)** | | **Variability*** | |
|  | Coral cells | Algae cells | Coral cells | Algae cells |
| - By Washing only (n=3) | 2.22E+05 (± 1.23E+05) | 1.38E+05 (± 1.23E+05) | ± 3.39E+05 | ± 1.49E+05 |
| - By mechanical scraping with razor blade or scalpel (n=27) | 1.78E+05 (± 2.23E+04) | 1.08E+06 (± 1.39E+05) | ± 1.84E+05 | ± 1.15E+06 |
| - By brushing: - Soft (paint brush, n=3) - Hard (toothbrush, n=9) | 9.51E+05 (± 5.48E+05)  1.09E+05 (± 3.74E+04) | 5.34E+04 (± 1.85E+04)  1.07E+06 (± 3.74E+04) | ± 1.51+06  ± 1.78E+05 | ± 5.11E+04  ± 1.78E+05 |
| - By Ca^2+^-, Mg^2+^-free seawater incubation: - 1h (n=4) - 2h (n=2) - 3h (n=2) - 24h (n=3) | 1.07E+07 (± 1.03E+06)  6.75E+06 (± 8.50E+05)  1.13E+06 (±7.95E+05)  1.65E+06 (± 9.53E+05) | 1.39E+06 (± 1.03E+06)  9.60E+05 (± 3.05E+04)  2.17E+05 (± 6.05E+04)  1.60E+05 (± 1.28E+05) | ± 3.28E+06  ± 1.91+06  ± 1.79E+06  ± 2.63E+06 | ± 3.28E+06  ± 6.86E+04  ± 1.36E+05  ± 3.52E+05 |
| **Enzyme digestion** | **Mean yield ± SE (cell/cm^2^)** | | **Variability*** | |
|  | Coral cells | Algae cells | Coral cells | Algae cells |
| - Enzyme degradation: - Scraping + trypsin (n=9) - Scraping + liberase (n=21) | 7.96E+04 (± 2.25E+04)  1.52E+05 (± 1.15E+04) | 2.20E+06 (± 8.29E+05)  4.51E+06 (± 4.58E+05) | ± 1.08E+05  ± 8.38E+04 | ± 3.96E+06  ± 3.34E+06 |
| **Cell sorting** | **Effectiveness** | | **Variability** | |
| - Percoll density gradient | Weak gradient with bleeding between layers; | | NA | |
| - FACS | Successfully separated the 2 main cell populations: symbiotic cells and asymbiotic cells; | | NA | |
| **Cell attachment** | **Attachment** | | **Variability*** | |
| - Untreated glass (n=3) - Collagen coated glass (n=3) - Tissue culture treated (TCT) plastic (n=3) - Collagen coated TCT plastic (n=3) | 60.64% (± 14.61%)  27.96% (± 12.70%)  39.57% (± 8.56%)  6.83% (± 1.21%) | | ± 28.64  ± 24.90  ±16.77  ± 2.37 | |

*Viability presented as 95% confidence interval of the mean
