# Supplementary material for: Applying model approaches in non-model systems: A review and case study on coral cell culture
Source: PLoS One. 2021 Apr 8;16(4):e0248953. doi: 10.1371/journal.pone.0248953 (PMC8031391; doi:10.1371/journal.pone.0248953)
Supplement: S2 Table — Algae cell and coral cell yields as a factor of dissociation method (washing, soft/hard brushing, mechanical scraping, or calcium-magnesium-free seawater incubation for 1 to 24 hours) and enzyme digestion (Trypsin or Liberase). (DOCX) [file pone.0248953.s002.docx]

**S.4. Table. Cell dissociation method comparison data.** Algae cell and coral cell yields as a factor of dissociation method (washing, soft/hard brushing, mechanical scraping, or calcium-magnesium-free seawater incubation for 1 to 24 hours) and enzyme degradation (Trypsin or Liberase).

| Algae | Mechanical  scraping | Soft  brushing | Hard  brushing | Washing  only | | Ca^2+^ Mg^2+^ free SW (1H) | Ca^2+^ Mg^2+^ free SW (2H) | Ca^2+^ Mg^2+^ free SW (3H) | Ca^2+^ Mg^2+^ free SW (24H) | Scraping + Liberase (20min) | Scraping + Trypsin  (10min) |
| --- | --- | --- | --- | --- | --- | --- | --- | --- | --- | --- | --- |
| n | 27 | 3 | 9 | 3 | | 4 | 2 | 2 | 3 | 21 | 9 |
|  | 1.53E+06 | 6.79E+04 | 8.50E+05 | 2.46E+05 | | 2.48E+06 | 9.90E+05 | 2.77E+05 | 4.15E+05 | 6.12E+06 | 2.08E+06 |
|  | 7.50E+05 | 1.67E+04 | 4.33E+05 | 8.29E+04 | | 7.55E+05 | 9.29E+05 | 1.56E+05 | 3.55E+04 | 4.70E+06 | 3.40E+06 |
|  | 3.50E+05 | 7.58E+04 | 1.42E+06 | 8.53E+04 | | 1.28E+06 |  |  | 3.09E+04 | 4.77E+06 | 1.48E+06 |
|  | 2.33E+05 |  | 2.17E+05 |  | | 1.05E+06 |  |  |  | 1.57E+06 | 8.33E+06 |
|  | 3.83E+05 |  | 5.83E+05 |  | |  |  |  |  | 5.88E+06 | 1.43E+06 |
|  | 4.83E+05 |  | 1.28E+06 |  | |  |  |  |  | 5.77E+06 | 1.58E+06 |
|  | 2.02E+06 |  | 3.52E+06 |  | |  |  |  |  | 6.68E+06 | 6.67E+04 |
|  | 5.33E+05 |  | 5.17E+05 |  | |  |  |  |  | 7.07E+06 | 8.33E+05 |
|  | 2.52E+06 |  | 8.00E+05 |  | |  |  |  |  | 4.83E+06 | 6.00E+05 |
|  | 1.43E+06 |  |  |  | |  |  |  |  | 1.38E+06 |  |
|  | 1.03E+06 |  |  |  | |  |  |  |  | 1.50E+06 |  |
|  | 1.17E+06 |  |  |  | |  |  |  |  | 8.02E+06 |  |
|  | 1.03E+06 |  |  |  | |  |  |  |  | 5.27E+06 |  |
|  | 9.33E+05 |  |  |  | |  |  |  |  | 5.88E+06 |  |
|  | 2.15E+06 |  |  |  | |  |  |  |  | 6.03E+06 |  |
|  | 2.48E+06 |  |  |  | |  |  |  |  | 4.43E+06 |  |
|  | 2.18E+06 |  |  |  | |  |  |  |  | 5.25E+06 |  |
|  | 1.37E+06 |  |  |  | |  |  |  |  | 3.07E+06 |  |
|  | 3.67E+05 |  |  |  | |  |  |  |  | 4.15E+06 |  |
|  | 1.33E+05 |  |  |  | |  |  |  |  | 1.88E+06 |  |
|  | 5.33E+05 |  |  |  | |  |  |  |  | 4.83E+05 |  |
|  | 1.05E+06 |  |  |  | |  |  |  |  |  |  |
|  | 4.83E+05 |  |  |  | |  |  |  |  |  |  |
|  | 6.00E+05 |  |  |  | |  |  |  |  |  |  |
|  | 5.50E+05 |  |  |  | |  |  |  |  |  |  |
|  | 9.67E+05 |  |  |  | |  |  |  |  |  |  |
|  | 1.98E+06 |  |  |  | |  |  |  |  |  |  |
| Coral | Mechanical  scraping | Soft  brushing | Hard  brushing | | Washing  only | Ca^2+^ Mg^2+^ free SW (1H) | Ca^2+^ Mg^2+^ free SW (2H) | Ca^2+^ Mg^2+^ free SW (3H) | Ca^2+^ Mg^2+^ free SW (24H) | Scraping + Liberase (20min) | Scraping + Trypsin  (10min) |
| n | 27 | 3 | 9 | | 3 | 4 | 2 | 2 | 3 | 21 | 9 |
|  | 2.50E+05 | 4.63E+05 | 1.83E+05 | | 3.08E+04 | 1.03E+07 | 5.90E+06 | 1.21E+06 | 3.56E+06 | 2.67E+05 | 0.00E+00 |
|  | 1.00E+05 | 3.44E+05 | 8.33E+04 | | 4.51E+05 | 7.96E+06 | 7.60E+06 | 1.04E+06 | 7.45E+05 | 1.33E+05 | 3.33E+04 |
|  | 1.00E+05 | 2.05E+06 | 3.67E+05 | | 1.83E+05 | 1.26E+07 |  |  | 6.48E+05 | 1.33E+05 | 3.33E+04 |
|  | 0.00E+00 |  | 5.00E+04 | |  | 1.19E+07 |  |  |  | 8.33E+04 | 1.67E+04 |
|  | 0.00E+00 |  | 1.00E+05 | |  |  |  |  |  | 1.67E+05 | 1.50E+05 |
|  | 5.00E+04 |  | 1.33E+05 | |  |  |  |  |  | 1.00E+05 | 1.83E+05 |
|  | 3.33E+04 |  | 5.00E+04 | |  |  |  |  |  | 2.50E+05 | 5.00E+04 |
|  | 5.00E+04 |  | 0.00E+00 | |  |  |  |  |  | 6.67E+04 | 1.50E+05 |
|  | 3.33E+04 |  | 1.67E+04 | |  |  |  |  |  | 1.33E+05 | 1.00E+05 |
|  | 1.00E+05 |  |  | |  |  |  |  |  | 2.17E+05 |  |
|  | 1.33E+05 |  |  | |  |  |  |  |  | 1.67E+05 |  |
|  | 1.33E+05 |  |  | |  |  |  |  |  | 2.00E+05 |  |
|  | 2.00E+05 |  |  | |  |  |  |  |  | 1.50E+05 |  |
|  | 2.17E+05 |  |  | |  |  |  |  |  | 1.33E+05 |  |
|  | 2.50E+05 |  |  | |  |  |  |  |  | 1.17E+05 |  |
|  | 1.50E+05 |  |  | |  |  |  |  |  | 1.17E+05 |  |
|  | 3.00E+05 |  |  | |  |  |  |  |  | 2.17E+05 |  |
|  | 1.17E+05 |  |  | |  |  |  |  |  | 1.17E+05 |  |
|  | 2.33E+05 |  |  | |  |  |  |  |  | 1.67E+05 |  |
|  | 4.00E+05 |  |  | |  |  |  |  |  | 1.33E+05 |  |
|  | 2.83E+05 |  |  | |  |  |  |  |  | 1.17E+05 |  |
|  | 2.33E+05 |  |  | |  |  |  |  |  |  |  |
|  | 2.50E+05 |  |  | |  |  |  |  |  |  |  |
|  | 2.83E+05 |  |  | |  |  |  |  |  |  |  |
|  | 2.67E+05 |  |  | |  |  |  |  |  |  |  |
|  | 2.17E+05 |  |  | |  |  |  |  |  |  |  |
|  | 4.33E+05 |  |  | |  |  |  |  |  |  |  |
