# Supplementary material for: Applying model approaches in non-model systems: A review and case study on coral cell culture
Source: PLoS One. 2021 Apr 8;16(4):e0248953. doi: 10.1371/journal.pone.0248953 (PMC8031391; doi:10.1371/journal.pone.0248953)
Supplement: S3 Table — Percent viability of coral cells dissociated using different methods (soft/hard brushing, mechanical scraping, or calcium-magnesium-free seawater incubation for 1 to 24 hours, and enzyme digestion). Each method was replicated at least three times. (DOCX) [file pone.0248953.s003.docx]

**S.5. Table. Overall cell viability immediately after dissociation, as a factor of dissociation method: data.** Percent viability of coral cells dissociated using different methods (soft/hard brushing, mechanical scraping, or calcium-magnesium-free seawater incubation for 1 to 24 hours, and enzyme digestion). Each method was replicated at least three times.

| Method | Replicate number | Viability (%) |
| --- | --- | --- |
| Soft_brushing | 1 | 35.41 |
| Soft_brushing | 2 | 41.42 |
| Soft_brushing | 3 | 36.02 |
| Hard_brushing | 1 | 54.30 |
| Hard_brushing | 2 | 46.77 |
| Hard_brushing | 3 | 59.83 |
| Mechanical_scraping | 1 | 59.37 |
| Mechanical_scraping | 2 | 51.64 |
| Mechanical_scraping | 3 | 53.63 |
| Ca_Mg_free_SW1H | 1 | 71.24 |
| Ca_Mg_free_SW1H | 2 | 65.30 |
| Ca_Mg_free_SW1H | 3 | 60.83 |
| Ca_Mg_free_SW1H | 4 | 77.79 |
| Ca_Mg_free_SW1H | 5 | 73.88 |
| Ca_Mg_free_SW1H | 6 | 73.43 |
| Ca_Mg_free_SW2H | 1 | 67.53 |
| Ca_Mg_free_SW2H | 2 | 54.77 |
| Ca_Mg_free_SW2H | 3 | 62.81 |
| Ca_Mg_free_SW3H | 1 | 59.95 |
| Ca_Mg_free_SW3H | 2 | 62.71 |
| Ca_Mg_free_SW3H | 3 | 59.87 |
| Ca_Mg_free_SW24H | 1 | 55.42 |
| Ca_Mg_free_SW24H | 2 | 52.08 |
| Ca_Mg_free_SW24H | 3 | 59.41 |
| Scraping_Liberase20min | 1 | 58.42 |
| Scraping_Liberase20min | 2 | 61.61 |
| Scraping_Liberase20min | 3 | 56.75 |
| Scraping_Trypsin10min | 1 | 50.98 |
| Scraping_Trypsin10min | 2 | 53.08 |
| Scraping_Trypsin10min | 3 | 52.63 |
