# Supplementary material for: Applying model approaches in non-model systems: A review and case study on coral cell culture
Source: PLoS One. 2021 Apr 8;16(4):e0248953. doi: 10.1371/journal.pone.0248953 (PMC8031391; doi:10.1371/journal.pone.0248953)
Supplement: S4 Table — Algae cell and coral host cell yields as a factor of the origin of cell counted: Initial cell dissociation performed by scraping (n = 18), remaining cells (on the skeleton) were dissociated using trypsin incubation for 1 hour (n = 9). (DOCX) [file pone.0248953.s004.docx]

| **Cell types** | **Scraped** | **Scraped + trypsin** |
| --- | --- | --- |
| Algae | 1.72E+06 | 1.78E+06 |
|  | 3.33E+05 | 3.68E+06 |
|  | 5.88E+06 | 2.82E+06 |
|  | 2.37E+06 | 2.08E+06 |
|  | 4.13E+06 | 2.57E+06 |
|  | 1.27E+06 | 1.42E+06 |
|  | 3.00E+05 | 2.20E+06 |
|  | 4.83E+05 | 3.22E+06 |
|  | 4.83E+05 | 3.55E+06 |
|  | 5.12E+06 |  |
|  | 4.32E+06 |  |
|  | 3.77E+06 |  |
|  | 1.83E+06 |  |
|  | 1.78E+06 |  |
|  | 2.70E+06 |  |
|  | 2.70E+06 |  |
|  | 4.42E+06 |  |
|  | 5.90E+06 |  |
| Coral | 0.00E+00 | 3.50E+05 |
|  | 0.00E+00 | 4.17E+05 |
|  | 4.67E+05 | 2.33E+05 |
|  | 6.67E+04 | 1.67E+05 |
|  | 3.17E+05 | 2.17E+05 |
|  | 2.17E+05 | 1.83E+05 |
|  | 6.67E+04 | 2.00E+05 |
|  | 0.00E+00 | 2.00E+05 |
|  | 1.83E+05 | 3.50E+05 |
|  | 7.83E+05 |  |
|  | 9.00E+05 |  |
|  | 2.00E+05 |  |
|  | 4.83E+05 |  |
|  | 3.00E+05 |  |
|  | 1.83E+05 |  |
|  | 3.17E+05 |  |
|  | 3.50E+05 |  |
|  | 4.33E+05 |  |

**S.6. Table. Cell dissociation method combination efficacy data.** Algae cell and coral host cell yields as a factor of the origin of cell counted: initial cell dissociation performed by scraping (n = 18), remaining cells (on the skeleton) were dissociated using trypsin incubation for 1 hour (n = 9).
