# Supplementary material for: Applying model approaches in non-model systems: A review and case study on coral cell culture
Source: PLoS One. 2021 Apr 8;16(4):e0248953. doi: 10.1371/journal.pone.0248953 (PMC8031391; doi:10.1371/journal.pone.0248953)
Supplement: S5 Table — Four substrates were tested for coral cell attachment: Tissue culture treated plastic (TCT), collagen coated TCT, glass and collagen coated glass. Cells were counted and the data is presented below (counts and percentages). (DOCX) [file pone.0248953.s005.docx]

| **Tissue culture treated (TCT) plastic** | | **Collagen coated TCT plastic** | | **Glass** | | **Collagen coated glass** | |
| --- | --- | --- | --- | --- | --- | --- | --- |
| Attachment (cell count) | Attachment (%) | Attachment (cell count) | Attachment (%) | Attachment (cell count) | Attachment (%) | Attachment (cell count) | Attachment (%) |
| 81,994.37 | 35.48 | 22,656.34 | 9.80 | 181,925.00 | 78.73 | 20,363.73 | 8.81 |
| 137,286.62 | 59.41 | 12,676.76 | 5.49 | 181,115.85 | 78.38 | 135,803.17 | 58.77 |
| 55,022.54 | 23.81 | 12,137.32 | 5.25 | 57,450.00 | 24.86 | 37,625.70 | 16.28 |

**S.7. Table.** **Cell attachment experiment data.** Four substrates were tested for coral cell attachment: tissue culture treated plastic (TCT), collagen coated TCT, glass and collagen coated glass. Cells were counted and the data is presented below (counts and percentages)
