# Supplementary material for: Applying model approaches in non-model systems: A review and case study on coral cell culture
Source: PLoS One. 2021 Apr 8;16(4):e0248953. doi: 10.1371/journal.pone.0248953 (PMC8031391; doi:10.1371/journal.pone.0248953)
Supplement: S6 Table — Average contamination free cell culture duration (days ± SE) as a factor of media type (F12, RPMI, DMEM), serum (0%, 5%, 10%) and antibiotic (Gentamicin, Anti-Anti: Antibiotic-Antimycotic, Anti-Anti + Gentamicin, Pen-Strep: Penicillin-Streptomycin). Thirty-six combinations [media + serum + antibiotic] tested with three replicates per combination. (DOCX) [file pone.0248953.s006.docx]

**S.8. Table. Coral cell culture contamination rate data.** Average contamination free cell culture duration (days ± SE) as a factor of media type (F12, RPMI, DMEM), serum (0%, 5%, 10%) and antibiotic (Gentamicin, Anti-Anti: Antibiotic-Antimycotic, Anti-Anti + Gentamicin, Pen-Strep: Penicillin-Streptomycin). Thirty-six combinations [media + serum + antibiotic] tested with three replicates per combination.

| **Sample ID** | **FBS (%)** | **MEDIA** | **ANTIBIOTIC** | **days** |
| --- | --- | --- | --- | --- |
| R101 | 0 | DMEM | G | 0 |
| R102 | 5 | DMEM | G | 2 |
| R103 | 10 | DMEM | G | 2 |
| R104 | 0 | RMPI | G | 0 |
| R105 | 5 | RMPI | G | 5 |
| R106 | 10 | RMPI | G | 5 |
| R107 | 0 | F12 | G | 0 |
| R108 | 5 | F12 | G | 5 |
| R109 | 10 | F12 | G | 5 |
| R110 | 0 | DMEM | AA+G | 0 |
| R111 | 5 | DMEM | AA+G | 5 |
| R112 | 10 | DMEM | AA+G | 5 |
| R113 | 0 | RMPI | AA+G | 0 |
| R114 | 5 | RMPI | AA+G | 5 |
| R115 | 10 | RMPI | AA+G | 1 |
| R116 | 0 | F12 | AA+G | 0 |
| R117 | 5 | F12 | AA+G | 2 |
| R118 | 10 | F12 | AA+G | 6 |
| R119 | 0 | DMEM | PS | 1 |
| R120 | 5 | DMEM | PS | 0 |
| R121 | 10 | DMEM | PS | 5 |
| R122 | 0 | RMPI | PS | 0 |
| R123 | 5 | RMPI | PS | 5 |
| R124 | 10 | RMPI | PS | 2 |
| R125 | 0 | F12 | PS | 0 |
| R126 | 5 | F12 | PS | 2 |
| R127 | 10 | F12 | PS | 2 |
| R128 | 0 | DMEM | AA | 0 |
| R129 | 5 | DMEM | AA | 5 |
| R130 | 10 | DMEM | AA | 5 |
| R131 | 0 | RMPI | AA | 0 |
| R132 | 5 | RMPI | AA | 6 |
| R133 | 10 | RMPI | AA | 1 |
| R134 | 0 | F12 | AA | 0 |
| R135 | 5 | F12 | AA | 5 |
| R136 | 10 | F12 | AA | 5 |
| R201 | 0 | DMEM | G | 0 |
| R202 | 5 | DMEM | G | 1 |
| R203 | 10 | DMEM | G | 2 |
| R204 | 0 | RMPI | G | 0 |
| R205 | 5 | RMPI | G | 1 |
| R206 | 10 | RMPI | G | 2 |
| R207 | 0 | F12 | G | 0 |
| R208 | 5 | F12 | G | 3 |
| R209 | 10 | F12 | G | 3 |
| R210 | 0 | DMEM | AA+G | 0 |
| R211 | 5 | DMEM | AA+G | 1 |
| R212 | 10 | DMEM | AA+G | 7 |
| R213 | 0 | RMPI | AA+G | 0 |
| R214 | 5 | RMPI | AA+G | 2 |
| R215 | 10 | RMPI | AA+G | 7 |
| R216 | 0 | F12 | AA+G | 0 |
| R217 | 5 | F12 | AA+G | 3 |
| R218 | 10 | F12 | AA+G | 3 |
| R219 | 0 | DMEM | PS | 1 |
| R220 | 5 | DMEM | PS | 8 |
| R221 | 10 | DMEM | PS | 8 |
| R222 | 0 | RMPI | PS | 0 |
| R223 | 5 | RMPI | PS | 2 |
| R224 | 10 | RMPI | PS | 3 |
| R225 | 0 | F12 | PS | 0 |
| R226 | 5 | F12 | PS | 3 |
| R227 | 10 | F12 | PS | 10 |
| R228 | 0 | DMEM | AA | 3 |
| R229 | 5 | DMEM | AA | 3 |
| R230 | 10 | DMEM | AA | 3 |
| R231 | 0 | RMPI | AA | 0 |
| R232 | 5 | RMPI | AA | 7 |
| R233 | 10 | RMPI | AA | 3 |
| R234 | 0 | F12 | AA | 0 |
| R235 | 5 | F12 | AA | 7 |
| R236 | 10 | F12 | AA | 7 |
| R301 | 0 | DMEM | G | 1 |
| R302 | 5 | DMEM | G | 1 |
| R303 | 10 | DMEM | G | 2 |
| R304 | 0 | RMPI | G | 0 |
| R305 | 5 | RMPI | G | 5 |
| R306 | 10 | RMPI | G | 1 |
| R307 | 0 | F12 | G | 0 |
| R308 | 5 | F12 | G | 3 |
| R309 | 10 | F12 | G | 2 |
| R310 | 0 | DMEM | AA+G | 0 |
| R311 | 5 | DMEM | AA+G | 2 |
| R312 | 10 | DMEM | AA+G | 1 |
| R313 | 0 | RMPI | AA+G | 0 |
| R314 | 5 | RMPI | AA+G | 5 |
| R315 | 10 | RMPI | AA+G | 3 |
| R316 | 0 | F12 | AA+G | 1 |
| R317 | 5 | F12 | AA+G | 1 |
| R318 | 10 | F12 | AA+G | 6 |
| R319 | 0 | DMEM | PS | 0 |
| R320 | 5 | DMEM | PS | 7 |
| R321 | 10 | DMEM | PS | 7 |
| R322 | 0 | RMPI | PS | 0 |
| R323 | 5 | RMPI | PS | 1 |
| R324 | 10 | RMPI | PS | 1 |
| R325 | 0 | F12 | PS | 1 |
| R326 | 5 | F12 | PS | 5 |
| R327 | 10 | F12 | PS | 5 |
| R328 | 0 | DMEM | AA | 7 |
| R329 | 5 | DMEM | AA | 7 |
| R330 | 10 | DMEM | AA | 7 |
| R331 | 0 | RMPI | AA | 0 |
| R332 | 5 | RMPI | AA | 1 |
| R333 | 10 | RMPI | AA | 1 |
| R334 | 0 | F12 | AA | 0 |
| R335 | 5 | F12 | AA | 7 |
| R336 | 10 | F12 | AA | 1 |
