# Supplementary material for: Applying model approaches in non-model systems: A review and case study on coral cell culture
Source: PLoS One. 2021 Apr 8;16(4):e0248953. doi: 10.1371/journal.pone.0248953 (PMC8031391; doi:10.1371/journal.pone.0248953)
Supplement: S7 Table — FBS: 0%, 5%, 10%. MEDIA: F12, RPMI, DMEM. ANTIBIOTIC: Gentamicin, Antibiotic-Antimycotic, Antibiotic-Antimycotic + Gentamicin, Penicillin-Streptomycin. (DOCX) [file pone.0248953.s007.docx]

**S.9. Table.** **Cell culture contamination variation ANCOVA results**. FBS: 0%, 5%, 10%. MEDIA: F12, RPMI, DMEM. ANTIBIOTIC: Gentamicin, Antibiotic-Antimycotic, Antibiotic-Antimycotic + Gentamicin, Penicillin-Streptomycin.

| Effect | df | F | p |
| --- | --- | --- | --- |
| FBS | 1 | 48.41 | 7.04E-10* |
| MEDIA | 2 | 2.074 | 0.13 |
| ANTIBIOTIC | 3 | 2.48 | 0.06 |
| MEDIA:ANTIBIOTIC | 6 | 1.62 | 0.15 |
| FBS:MEDIA | 2 | 1.25 | 0.29 |
| FBS:ANTIBIOTIC | 3 | 1.08 | 0.36 |
| FBS:MEDIA:ANTIBIOTIC | 6 | 0.48 | 0.82 |
