# Supplementary material for: Applying model approaches in non-model systems: A review and case study on coral cell culture
Source: PLoS One. 2021 Apr 8;16(4):e0248953. doi: 10.1371/journal.pone.0248953 (PMC8031391; doi:10.1371/journal.pone.0248953)
Supplement: S8 Table — Percent viability of coral cells dissociated using calcium-magnesium-free seawater incubation for 1 hour and grown for 7 days in growth media (15% DMEM + 10% FBS + 1% Penicillin-Streptomycin + 74% filtered artificial sterile seawater, media replenished on days 2 and 5, n = 3). (DOCX) [file pone.0248953.s008.docx]

**S. 10. Table. Cell viability (%) after 7 days culture: data.** Percent viability of coral cells dissociated using calcium-magnesium-free seawater incubation for 1 hour and grown for 7 days in growth media (15% DMEM + 5% FBS + 1% Antibiotic-Antimycotic + 79% filtered artificial sterile seawater, media replenished on days 2 and 5, n=3).

| **Dissociation method** | **replicate** | **day** | **live cells/cm^2** | **dead cells/cm^2** | **total cells/cm^2** | **%viability** |
| --- | --- | --- | --- | --- | --- | --- |
| **CaMg free SW 1H** | 1 | 0 | 1.19E+06 | 3.40E+05 | 1.53E+06 | 77.8 |
| **CaMg free SW 1H** | 2 | 0 | 7.70E+05 | 2.72E+05 | 1.04E+06 | 73.9 |
| **CaMg free SW 1H** | 3 | 0 | 1.01E+06 | 3.66E+05 | 1.38E+06 | 73.4 |
| **CaMg free SW 1H** | 1 | 2 | 1.07E+06 | 4.44E+05 | 1.51E+06 | 70.6 |
| **CaMg free SW 1H** | 2 | 2 | 4.26E+05 | 2.72E+05 | 6.99E+05 | 61.0 |
| **CaMg free SW 1H** | 3 | 2 | 4.65E+05 | 1.72E+05 | 6.37E+05 | 73.0 |
| **CaMg free SW 1H** | 1 | 5 | 5.78E+04 | 9.49E+04 | 1.53E+05 | 37.8 |
| **CaMg free SW 1H** | 2 | 5 | 9.76E+04 | 1.39E+05 | 2.37E+05 | 41.3 |
| **CaMg free SW 1H** | 3 | 5 | 8.66E+04 | 9.63E+04 | 1.83E+05 | 47.4 |
| **CaMg free SW 1H** | 1 | 7 | 9.63E+03 | 3.85E+04 | 4.81E+04 | 20.0 |
| **CaMg free SW 1H** | 2 | 7 | 3.44E+04 | 2.75E+04 | 6.19E+04 | 55.6 |
| **CaMg free SW 1H** | 3 | 7 | 1.93E+04 | 3.03E+04 | 4.95E+04 | 38.9 |
