# Supplementary material for: Applying model approaches in non-model systems: A review and case study on coral cell culture
Source: PLoS One. 2021 Apr 8;16(4):e0248953. doi: 10.1371/journal.pone.0248953 (PMC8031391; doi:10.1371/journal.pone.0248953)
Supplement: S9 Table — (DOCX) [file pone.0248953.s009.docx]

**S.11. Table. Composition of different growth media used in this study**

| **Component** | **F-12K** | **RPMI 1640** | **DMEM** |
| --- | --- | --- | --- |
|  | **Concentration (mg/L)** | | |
| **Inorganic salts:** |  |  |  |
| CaCl_2_•2H_2_O | 135.24 | 0 | 0 |
| CaCl_2_ (anhydrous) | 0 | 0 | 200 |
| CuSO_4_•5H_2_O | 0.0025 | 0 | 0 |
| Fe3NO_3_•9H_2_O | 0 | 0 | 0.1 |
| FeSO_4_•7H_2_O | 0.834 | 0 | 0 |
| MgCl_2_•6H_2_O | 105.72 | 0 | 0 |
| MgSO_4_ (anhydrous) | 192.64 | 0 | 97.67 |
| MgSO_4_•7H_2_O | 0 | 100 | 0 |
| KCl | 283.29 | 400 | 400 |
| KH_2_PO_4_ (anhydrous) | 58.52 | 0 | 0 |
| NaHCO_3_ | 1500 | 2000 | 3700 |
| Na_2_HPO_4_ (anhydrous) | 115,02 | 0 | 0 |
| Na_2_HPO_4_•H_2_O | 0 | 151 | 125 |
| NaCl | 7597.2 | 6000 | 6400 |
| ZnSO_4_•7H_2_O | 0.144 | 0 | 0 |
| Ca(NO3)2•4H2O | 0 | 100 | 0 |
| **Amino Acids:** |  | | |
| L-Arginine | 421.4 | 200 | 0 |
| L-Alanine | 17.82 | 0 | 0 |
| L-Asparagine | 30.20 | 50 | 0 |
| L-Asparagine•HCl | 0 | 0 | 84 |
| L-Aspartic Acid | 26.62 | 20 | 0 |
| L-Cysteine•HCl•H_2_O | 70.24 | 0 | 0 |
| L-Cystine•2HCl | 0 | 65 | 63 |
| L-Glutamic Acid | 29.42 | 20 | 0 |
| L-Glutamine | 292.2 | 300 | 584 |
| Glycine | 15.01 | 0 | 30 |
| L-Histidine•HCl•H_2_O | 41.91 | 0 | 42 |
| L-Histidine | 0 | 15 | 0 |
| L-Hydroxyproline | 0 | 20 | 0 |
| L-Isoleucine | 7.82 | 50 | 105 |
| L-Leucine | 26.24 | 50 | 105 |
| L-Lysine•HCl | 73.04 | 40 | 146 |
| L-Methionine | 89.95 | 15 | 30 |
| L-Phenylalanine | 9.91 | 15 | 66 |
| L-Proline | 69.06 | 20 | 0 |
| L-Serine | 21.02 | 30 | 42 |
| L-Threonine | 23.83 | 20 | 95 |
| L-Tryptophan | 4.08 | 5 | 16 |
| L-Tyrosine | 10.87 | 0 | 0 |
| L-Tyrosine•2Na•2H_2_O | 0 | 29 | 104 |
| L-Valine | 23.42 | 20 | 94 |
| **Vitamins:** |  |  |  |
| D-Biotin | 0.0733 | 0.20 | 0 |
| Choline Chloride | 13.96 | 3 | 4 |
| D-Calcium Pantothenate | 0 | 0.25 | 4 |
| Folic Acid | 1.32 | 1 | 4 |
| Hypoxanthine | 4.08 | 0 | 0 |
| i-Inositol | 0 | 35 | 7.2 |
| myo-Inositol | 18.02 | 0 | 0 |
| Niacinamide | 0 | 1 | 4 |
| Nicotinamide | 0.0366 | 0 | 0 |
| Para-Amonibenzoic Acid | 0 | 1 | 0 |
| D-Pantothenic Acid (hemicalcium) | 0.477 | 0 | 0 |
| Putrescine•2HCl | 0.322 | 0 | 0 |
| Pyridoxine•HCl | 0.0617 | 1 | 4 |
| Riboflavin | 0.0376 | 0.20 | 0.4 |
| Thiamine•HCl | 0.337 | 1 | 4 |
| Thymidine | 0.727 | 0 | 0 |
| Vitamin B-12 | 1.355 | 0.005 | 0 |
| **Other:** |  |  |  |
| D-Glucose | 1260 | 2000 | 4500 |
| Glutathione (reduced) | 0 | 1 | 0 |
| Phenol Red, Sodium salt | 3.32 | 5 | 15 |
| Sodium Pyruvate | 220 | 0 | 110 |
| Lipoic Acid | 0.21 | 0 | 0 |
