# Supplementary material for: Applying model approaches in non-model systems: A review and case study on coral cell culture
Source: PLoS One. 2021 Apr 8;16(4):e0248953. doi: 10.1371/journal.pone.0248953 (PMC8031391; doi:10.1371/journal.pone.0248953)
Supplement: S1 File — DNA sequence extracted from corals used for this study and link to associated protocol repository. (DOCX) [file pone.0248953.s010.docx]

**S.1. *Pocillopora acuta* sequence and sequencing protocol.**

>HAQ01_210112.ab1

NNNNNNNNNNNNTCNTCNTNCACNCTAGGNNCGACTANNGTCGAGCCAACTACCTCATCAATTTCTACTTTGTTTTCTCT

TAAAACCCCAATTTCTACCTTGTTTTCTCTTAAAGCCTCACCTTGATTTATACTGGCTCATCGGCCACCCCTTGAAGCAC

CTGGAGGTGTTTCTACCTTTATTTCACTTAAAGTTTGGTCTTGGCCTCTCCTCTCTATTAATGAATCACTTAATACATTT

CTTATATTACCACTTGGAGCGCCCCTATTTACACTTTCTGGTGTAAGTGTACTTGTACTCCCCCCTAAAAAAGCCACATC

TTCAAATTGATCTCCTAAACTATTCTCAACACCTTCTTGATTAGACCGATTATCCAAATAACCACGCCGCTCAACATGTA

GATTCACCCCCCTTACGGGGACCCTTCGCCCTTCCAATTCTATAAAAACAACATCCACAGACTGTTCAGACTCTTGATTC

ACTTGTACCCCAACATCATTTGGTCCTTCAGTTGAATCTAGATTCACTTGTACCCCAACATTACGAGCAATAGAATGCTG

GCGCACATAATTAAAATAAGCTCAAGGATGGACCCTCTCTGCACCGTCTATGCCCATTTGGTGCCTTAACCAGGCTTGAG

AAGAACTACCATATCACCCAATACCAAATCCATAATTCACCGTTAACGATATACCTACAATTGTGCATAAAAAAATTTCT

ACGGCTGTCATTTTTAATGTAAAAAAAATTGTATCTGTTAAATAAATTTGTACCAACAGACAAAACACATAGGCCTGTAT

TATTGCAACTGCCACCTCTAATAGCGTAATAAACACCATTATAAACATTGCAAATTTAAAAAACCAGACCAAATCCTGNC

NAAAATTGCAAATAAAAGATGCCCNGGCAGAAAGATTTGCTGCTAAACGAANNGCCNNAAA

Sequencing protocol: [Sampling, Extraction, and mtORF Amplification and Sanger Sequencing Prep of 2 Aquarium Corals – M.E. Schedl's Putnam Lab Open Lab Notebook – Notebook of a Lab Manager for the Putnam Lab](https://meschedl.github.io/MESPutnam_Open_Lab_Notebook/AQ-mtORF/)

<https://meschedl.github.io/MESPutnam_Open_Lab_Notebook/AQ-mtORF/>
