# Supplementary material for: Applying model approaches in non-model systems: A review and case study on coral cell culture
Source: PLoS One. 2021 Apr 8;16(4):e0248953. doi: 10.1371/journal.pone.0248953 (PMC8031391; doi:10.1371/journal.pone.0248953)
Supplement: S2 File — Protocols used for coral cell dissociation, attachment, culture, count and separation. (DOCX) [file pone.0248953.s011.docx]

**S. 3. Cell dissociation protocols and protocol repository**

<https://github.com/thesyntheticcoral/SynCoral_Protocols/tree/master/Culturing>

There are multiple options for isolation and incubation techniques.

**Table of Contents**

- [Materials](https://github.com/thesyntheticcoral/SynCoral_Protocols/blob/master/Culturing/Cell_Isolation_Protocol.md#Materials)
- [Culture-Media-Reagents-And-Recipes](https://github.com/thesyntheticcoral/SynCoral_Protocols/blob/master/Culturing/Cell_Isolation_Protocol.md#Culture-Media-Reagents-And-Recipes)
- [Dissociation](https://github.com/thesyntheticcoral/SynCoral_Protocols/blob/master/Culturing/Cell_Isolation_Protocol.md#Isolation)
- [Mechanical Scraping](https://github.com/thesyntheticcoral/SynCoral_Protocols/blob/master/Culturing/Cell_Isolation_Protocol.md#MechanicalScraping)
- [Toothbrush](https://github.com/thesyntheticcoral/SynCoral_Protocols/blob/master/Culturing/Cell_Isolation_Protocol.md#Toothbrush)
- [Paintbrush](https://github.com/thesyntheticcoral/SynCoral_Protocols/blob/master/Culturing/Cell_Isolation_Protocol.md#Paintbrush)
- [Enzyme](https://github.com/thesyntheticcoral/SynCoral_Protocols/blob/master/Culturing/Incubation) degradation
- [Trypsin](https://github.com/thesyntheticcoral/SynCoral_Protocols/blob/master/Culturing/Cell_Isolation_Protocol.md#Trypsin)
- [Liberase](https://github.com/thesyntheticcoral/SynCoral_Protocols/blob/master/Culturing/Cell_Isolation_Protocol.md#Liberase)
- [Incubation-Conditions](https://github.com/thesyntheticcoral/SynCoral_Protocols/blob/master/Culturing/Cell_Isolation_Protocol.md#Incubation-Conditions)
- [Viability](https://github.com/thesyntheticcoral/SynCoral_Protocols/blob/master/Culturing/Viability)
- Cell Attachment
- Cell Populations Separation
- Density Gradient
- Fluorescent Activated Cell Separation
- [General-Notes](https://github.com/thesyntheticcoral/SynCoral_Protocols/blob/master/Culturing/General-Notes)
- [References](https://github.com/thesyntheticcoral/SynCoral_Protocols/blob/master/Culturing/Cell_Isolation_Protocol.md#References)

**A.** **Materials**

- 20 gallon Carboy
  - Distilled water
  - 70% ethanol
- Medical grade toenail clippers
- Tweezers
- Toothbrush
- Paintbrush
- Scraper tool (surgical hook, sterile scalpel)
- Sterile filtered artificial seawater (regular and CaMg free)
- [Antibiotics-antimycotics solution Thermofisher #15240062](https://www.thermofisher.com/order/catalog/product/15240062#/15240062)
- Sterile plastic petri dish
- [Trypsin Fishersci #25300054](https://www.fishersci.com/shop/products/trypsin-05-edta/25300054)
- [Liberase solution with Collagenase Millipore Sigma #5401119001](https://www.sigmaaldrich.com/catalog/product/roche/libtmro?lang=en&region=US&gclid=Cj0KCQjwpNr4BRDYARIsAADIx9zfc5YGD6_945gvdyfUuAw24tSf-Xyaf-Xb_pm_QNG67XttLhyo7hMaAtpPEALw_wcB)
- Conical tube (15 mL)
- Centrifuge (can reach 1200 rpm)
- Shaking incubator (25 C, 30 rpm)
- Incubator [Thermo Scientific PR505755R Refrigerated Incubator, 20 cu ft, 120 VAC](https://www.coleparmer.com/i/thermo-scientific-pr505755r-refrigerated-incubator-20-cu-ft-120-vac/3935020)
- [Aqua Illumination Prime 16HD light](https://www.aquaillumination.com/products/prime)
- [Neptune Systems New APEX Wi-Fi Controller System #NS1191](https://www.marinedepot.com/neptune-systems-new-apex-wi-fi-controller-system)
- Relative humidity meter, example [ThermoPro TP55 Digital Hygrometer Indoor Thermometer Humidity Gauge with Jumbo Touchscreen and Backlight Temperature Humidity Monitor](https://www.amazon.com/gp/product/B06XTPTG1J/ref=ppx_yo_dt_b_asin_title_o00_s00?ie=UTF8&psc=1)
- Hotplate
- Pipette (P200, P20)
- [Trypan blue stain Millepore sigma #T8154](https://www.sigmaaldrich.com/catalog/product/sigma/t8154?lang=en&region=US)
- Hemocytometer
- Eppendorf tube

**B.** **Culture-Media-Reagents-And-Recipes**

**How to make coral culture media**

- 15 mL [Dulbecco's Modified Eagle Medium (DMEM) Fisher scientific #SH3024301](https://www.fishersci.com/shop/products/hyclone-classical-liquid-media-dulbeccos-modified-eagles-medium-dmem-high-with-l-glutamine-sodium-pyruvate-500ml/sh3024301)
- 10 mL [Fetal Bovine Serum (FBS) Thermofisher Scientific #26140087](https://www.thermofisher.com/order/catalog/product/26140087#/26140087)
- 1 mL [Antibiotics-antimycotics solution Thermofisher #15240062](https://www.thermofisher.com/order/catalog/product/15240062#/15240062)
- 0.5 mL [Gentamicin Thermofisher #15710064](https://www.thermofisher.com/order/catalog/product/15710064#/15710064)
- 74 mL artificial seawater (recipe below)
  - NOTE: Filter all ingredients using a Corning vacuum filter/storage bottle system with 0.2 μm pore size membrane.

**How to make bulk artificial seawater**

- Pour out salt from [Instant Ocean Reef crystals Reef Salt](https://www.amazon.com/Instant-Ocean-Reef-Crystals-Aquariums/dp/B000HCLNQG/ref=sr_1_1_sspa?crid=RF68CI3H2ZR1&dchild=1&keywords=instant+ocean+reef+crystals&qid=1596466354&s=pet-supplies&sprefix=instant+o%2Cpets%2C169&sr=1-1-spons&psc=1&spLa=ZW5jcnlwdGVkUXVhbGlmaWVyPUFPSFRUQjRUOEhBRk8mZW5jcnlwdGVkSWQ9QTAwOTEzNjgxRTlLNTlCVUg1WUJZJmVuY3J5cHRlZEFkSWQ9QTA5OTYxNDgzMVlTMkJWM1pGWk1OJndpZGdldE5hbWU9c3BfYXRmJmFjdGlvbj1jbGlja1JlZGlyZWN0JmRvTm90TG9nQ2xpY2s9dHJ1ZQ=)
- Add salt to 20 gallon Carboy
- Add 10 gal of distilled water to carboy (with salt)
- Shake Carboy with salt and distilled water thoroughly
  - NOTE: salt needs to be dissolved before use
  - NOTE: can use a (clean) aquarium pump to help dissolve salt

**How to make CaMg free seawater**

Lewinski Lab calcium-magnesium free artificial seawater mixture:

- 23 g/L NaCl
- 0.763 g/L KCl
- 3 g/L NaSO4
- 0.25 g/L NaHCO3

**C.** **Isolation**

1. Disinfect clippers and tweezers with 70% ethanol.
2. Remove coral fragment from tank and place in glass crystallizing dish containing tank water.
3. Cut coral nubbin (0.3-0.5 cm long) from fragment using sterilized clippers.
4. Using tweezers, carefully bring nubbin into the biosafety cabinet and place in a sterile plastic petri dish.
5. Rinse nubbin 3 times with sterile filtered artificial seawater in the petri dish.
6. Transfer rinsed nubbin to a different sterile plastic petri dish containing 5 mL of sterile filtered calcium-magnesium free artificial seawater.

**1.** **Mechanical Scraping**

- **1.1** Mechanically scrape nubbin using a sterile scalpel or surgical hook.
  - a. NOTE: Need to try scraping polyps only first then coenosarc as a means of separating cell types.
  - b. NOTE: Mechanical scrapping does not remove all tissue. Need to try enzymatic digestion of residual tissue from skeleton.
- **1.2** Transfer water containing detached tissue and cells to a 15 mL conical tube. Rinse petri dish with 5 mL of sterile filtered calcium-magnesium free artificial seawater to collect any tissue and cells that did not transfer. Centrifuge at 1200 rpm for 3 minutes. A pellet should be visible at the bottom of the conical tube.
- **1.3** Aspirate the supernatant and resuspend with 5 mL of coral cell culture media.
  - a. NOTE: If counting the collected cells, use a smaller volume (2 mL) to achieve a higher cell concentration for counting.
  - Lewinski lab coral culture media recipe here: [**Coral-Culture-Media-Reagents**](https://github.com/thesyntheticcoral/SynCoral_Protocols/blob/master/Culturing/Cell_Isolation_Protocol.md#Coral-Culture-Media-Reagents)

**2.** **Toothbrush**

- **2.1** Mechanically scrape nubbin using a toothbrush
  - a. NOTE: Need to try scraping polyps only first then coenosarc as a means of separating cell types.
- **2.2** Transfer water containing detached tissue and cells to a 15 mL conical tube and centrifuge at 1200 rpm for 3 minutes. A pellet should be visible at the bottom of the conical tube.
- **2.3** Aspirate the supernatant and resuspend with 5 mL of coral cell culture media.
  - Lewinski lab coral culture media recipe here: [**Coral-Culture-Media-Reagents**](https://github.com/thesyntheticcoral/SynCoral_Protocols/blob/master/Culturing/Cell_Isolation_Protocol.md#Coral-Culture-Media-Reagents)

**3.** **Paintbrush**

- **3.1** Mechanically scrape nubbin using a paintbrush
  - a. NOTE: Need to try scraping polyps only first then coenosarc as a means of separating cell types.
- **3.2** Transfer water containing detached tissue and cells to a 15 mL conical tube and centrifuge at 1200 rpm for 3 minutes. A pellet should be visible at the bottom of the conical tube.
- **3.3** Aspirate the supernatant and resuspend with 5 mL of coral cell culture media.
  - Lewinski lab coral culture media recipe here: [**Coral-Culture-Media-Reagents**](https://github.com/thesyntheticcoral/SynCoral_Protocols/blob/master/Culturing/Cell_Isolation_Protocol.md#Coral-Culture-Media-Reagents)

**4.** **Ca-Mg-free-seawater**

- **4.1** Incubate nubbin at 25°C for 1-3 hours in shaking incubator (rpm 30), in filtered, calcium and magnesium free, artificial seawater supplemented with 3% [Antibiotics-antimycotics solution Thermofisher #1524006](https://www.thermofisher.com/order/catalog/product/15240062#/15240062) solution.
- **4.2** Using tweezers, transfer nubbin to a sterile Eppendorf tube and rinse nubbin using 2 mL of filtered, calcium and magnesium free, artificial seawater supplemented with 3% [Antibiotics-antimycotics solution Thermofisher #1524006](https://www.thermofisher.com/order/catalog/product/15240062#/15240062) solution.
  - a. NOTE: An incubation of 1 hour yields on the order of 10^6 cells.
  - b. NOTE: A 3 hour incubation time does not collect all tissue. Incubation for 2 days results in significantly more tissue dissociated.

**5** **Trypsin**

- **5.1**. Transfer water containing detached tissue and cells to a conical tube (15 mL) and centrifuge at 1200 rpm for 3 minutes.
  - NOTE: A pellet should be visible at the bottom of the conical tube.
- **5.2** Aspirate the supernatant and add 2 mL of [Trypsin fishersci #25300054](https://www.fishersci.com/shop/products/trypsin-05-edta/25300054) to resuspend the pellet.
  - NOTE: for different trypsin concentrations: 1 mL of 0.25% trypsin stock solution + 1 mL of filtered artificial seawater (no [HEPES Thermofisher #15630106](https://www.thermofisher.com/us/en/home/life-science/cell-culture/mammalian-cell-culture/reagents/hepes.html)) to make 0.125% trypsin solution. 1 mL of 0.05% trypsin stock solution + 1 mL of filtered artificial seawater (no [HEPES Thermofisher #15630106](https://www.thermofisher.com/us/en/home/life-science/cell-culture/mammalian-cell-culture/reagents/hepes.html)) to make 0.025% trypsin solution.
    - NOTE: Both [trypsin] work and we need to measure the cell concentrations dissociated resulting from the different concentrations.
- **5.3** Incubate the resuspended cell mixture for 10 minutes at 25°C then check for cell separation (e.g. primarily individual cells in suspension, little to no large cell aggregates).
- **5.4** Add 3 mL of coral cell media to neutralize trypsin then centrifuge cells at 1200 rpm for 3 minutes.
- **5.5** Aspirate the supernatant and resuspend with 5 mL of coral cell culture media.
  - NOTE: Lewinski lab coral culture media recipe here: [**Coral-Culture-Media-Reagents**](https://github.com/thesyntheticcoral/SynCoral_Protocols/blob/master/Culturing/Cell_Isolation_Protocol.md#Coral-Culture-Media-Reagents)

**6.** **Liberase**

- **6.1**. Transfer water containing detached tissue and cells to a conical tube (15 mL) and centrifuge at 1200 rpm for 3 minutes.
  - NOTE: A pellet should be visible at the bottom of the conical tube.
- **6.2** Aspirate the supernatant 2 mL 0.05% Liberase.
  - NOTE: to make liberase stock solution, add 1 mL filtered artificial seawater to 2.5 mg [Liberase Millipore Sigma #5401119001](https://www.sigmaaldrich.com/catalog/product/roche/libtmro?lang=en&region=US&gclid=Cj0KCQjwpNr4BRDYARIsAADIx9zfc5YGD6_945gvdyfUuAw24tSf-Xyaf-Xb_pm_QNG67XttLhyo7hMaAtpPEALw_wcB). To reach the 0.05% liberase concentration used for the digestion, add 4 mL of additional filtered artificial seawater to 1 mL of stock liberase solution.
- **6.3** Incubate the resuspended cell mixture for 20 minutes at 25°C then check for cell separation (e.g. primarily individual cells in suspension, little to no large cell aggregates).
- **6.4** Add 3 mL of coral cell media to neutralize trypsin then centrifuge cells at 1200 rpm for 3 minutes.
- **6.5** Aspirate the supernatant and resuspend with 5 mL of coral cell culture media.
  - NOTE: Lewinski lab coral culture media recipe here: [**Coral-Culture-Media-Reagents**](https://github.com/thesyntheticcoral/SynCoral_Protocols/blob/master/Culturing/Cell_Isolation_Protocol.md#Coral-Culture-Media-Reagents)

**D.** **Incubation**

- **For mechanical dissociation with a medical scalpel, toothbrush, or paintbrush**: timing for incubation period undetermined as of 07282020

**E.** **Incubation-Conditions**

- Temperature: 25°C using a hotplate with a beaker of water
  - NOTE: keep relative humidity above 50% but below 80%. Need to modify procedure to control RH in small plexiglass incubator
- Light levels: TBD (no initial differences observed with or without light)
  - NOTE: use [Aqua Illumination Prime 16HD light](https://www.aquaillumination.com/products/prime) and monitor conditions using [Neptune Systems New APEX Wi-Fi Controller System #NS1191](https://www.marinedepot.com/neptune-systems-new-apex-wi-fi-controller-system)
  - NOTE: Can try 24 hr light cycle or 12:12 (light:dark).

**F.** **Viability**

9. Transfer 100 uL of the cell suspension to an Eppendorf tube and add 10 uL of trypan blue stain. Mix the cell suspension with the dye.

- a. NOTE: Trypan blue is a mutagen – handle with care and dispose contaminated items in biohazard waste!

1. Transfer 10 uL of the cell suspension to a hemocytometer and count the number of algae cells and coral cells noting the number of each cell type that stain blue.
2. Count at least three 1 mm x 1 mm squares per hemocytometer side to obtain six measurements in one experiment.
3. Report the following data:

| **Cell type** | **Total count** | **Dead count** |
| --- | --- | --- |
| Algae |  |  |
| Coral |  |  |

1. Calculate the cell solution concentration. C = c/d/A*f

- C = concentration (cells/mL)
- c = average number of cells counted (cells)
- d = depth of hemocytometer chamber (0.1 mm)
- A = area of hemocytometer counted (mm^2)
- f = conversion factor (1000 mm^3/mL)

1. Securely cap Eppendorf tube and dispose of trypan blue dyed cell suspension into biohazard waste.

**G. Cell Attachment**

- Collagen Coating Solution Protocol (https://www.sigmaaldrich.com/catalog/product/sigma/12550?lang=en&region=US)

The coating concentration is 1 ml per 10 cm^2^ surface area of the cell culture ware: 0.113 mL for coating a 12 mm diameter glass coverslip (2.5 ml for coating a T-25 flask or a 60 mm tissue culture dish;  7.5 ml for coating a T-75 flask or a 100 mm tissue culture dish). Add collagen coating solution to the substrate and rock gently to distribute evenly. Let sit for at room temperature overnight. Remove coating solution by aspiration in a sterile hood. The substrate is ready to use immediately, alternatively, it can be stored at 4°C for up to two weeks.

- Attachment test:

1.      Coat the substrate to test following to step outlined above. For plastic: directly coat wells of a multi-well plate. For glass: coat a glass coverslip placed inside a well of a multi-well plate.

2.      Dissociate cells using the Ca-Mg free seawater incubation (1H) method described previously and prepare the cell suspension.

3.     Count cells following the method described previously

4. Add 0.5 mL of cell suspension + 0.5 mL of media to each well and incubate at 25°C for 24H.

5. After 24H, vigorously mix the solution in each well using a pipette to dislodge unattached cells.

6. Add 0.5 mL of DAPI working solution ( DAPI 1 mg [Millipore Sigma cat. no. D9542]; Stock solution = 1 mg/mL DAPI in DI water; Working solution = 1 μg/ml DAPI in PBS) to each well to stain cells.

7. Count cells.

**H. Cell Populations Separation**

- Density Gradient: the protocol used was adapted from Downs et al., 2009

1. Dissociate cells using the Ca-Mg free seawater incubation (1H) method described previously and prepare the cell suspension;

2. Prepare 90, 80, 70, 60, 50, 40, 30, 20, 10, 5% Percoll solutions by mixing 100% Percoll with the appropriate volume of filtered artificial seawater;

3. Refrigerate each of the eleven solutions (5-100%) for at least 30 minutes before construction of the Percoll gradient;

4. In a 15 mL conical tube, add 1 mL per Percoll concentration starting with 100% and working towards 10%;

5. Add 2 mL of coral cell sample to the top of the Percoll gradient;

6. Centrifuge tube at 400g (1460 rpm for swing bucket rotor) for 10 minutes.

Note: Ficoll 400 and PEG 8000 were used as additives by Downs et al. (2009) but made no difference on the outcome of our Percoll Step-Gradient cell separation tests.

- Fluorescence Activated Cell Separation

1. Dissociate cells using the Ca-Mg free seawater incubation (1H) method described previously and prepare the cell suspension;

2. Transfer cel suspension to a conical tube and centrifuge at 1200 rom for 3 minutes to pellet cells;

3. Remove supernatant and resuspend in 3 mL od DAPI working solution ( DAPI 1 mg [Millipore Sigma cat. no. D9542]; Stock solution = 1 mg/mL DAPI in DI water; Working solution = 1 μg/ml DAPI in PBS) – Note: this is a short cut to stain while counting. Separate steps if anticipate cell counting will take longer than 15 minutes;

4. Count cells then centrifuge and resuspend cell pellet in volume of 1X PBS for cell concentration of 1-2 x10^6^ cells/mL;

5. Aliquot cells as needed;

6. Filter 1 mL of cells through cell strainer into FACS tube (Corning Falcon test tube with cell strainer snap cap [Fisher Scientific cat. No. 08-771-23];

7. Store FACS tubes at 25°C until analysis (within 2H).

8. Cell sorting using FACS (based on Rosental et al. 2017):

- FACS machine used: BD FACSAria™ II High-Speed Cell Sorter with BD FACSDiva software;

- Initial gating based on forward (FSC) and side scattered (SSC) light to focus on cell signal versus cell debris. Subsequent gating aimed at separating coral host cells (green autofluorescence) from symbiotic algae cells (red autofluorescence);

- Channels used (adapted from Rosental et al. 2017): autofluorescence 640nm (780/60BP) versus DAPI 405nm (450/50BP) and autofluorescence 488 (710/50BP) versus DAPI 405nm (450/50BP).

**I. General-Notes**

1. When used 1 mL PenStrep, microorganism overgrowth observed on day 2 post-dissociation.
2. When scraping off cells and can use original skeleton and place in media. sometimes has less gunk.
3. NOTE: currently using cell viability (% alive & % dead) for protocol assessment. In the future we could incorporate metrics beyond cell viability (e.g. cell cycle, oxidative stress, DNA damage).
4. other relevant Antibiotics: Gentamicin sulfate (Sigma, Cat# G1914), Neomycin sulfate (Sigma, Cat# PHR1491), Tobramycin sulfate (Sigma, Cat# T1783), Kanamycin sulfate (Sigma, Cat# 60615), Levofloxacin (Sigma, Cat# 28266-10G-F), Cefepime hydrochloride (Cat#PHR1763-1G, sigma).
